# Tiny stresses are capable of triggering earthquakes and tremors in Arunachal Himalaya

**DOI:** 10.1038/s41598-023-49068-3

**Published:** 2023-12-14

**Authors:** Tony Saini, Abhey Ram Bansal, N. Purnachandra Rao, Rajat Pasricha, Venkatesh Vempati

**Affiliations:** 1 CSIR-National Geophysical Research Institute, Uppal Road, Hyderabad, 500007 India; 2 Academy of Scientific and Innovative Research (AcSIR), Ghaziabad, 201002 India; 3 Ministry of Earth Sciences (MoES) Prithvi Bhawan, New Delhi, 110003 India

**Keywords:** Natural hazards, Solid Earth sciences

## Abstract

The Arunachal Himalaya has been hosting some notable events in the recent past. The tectonic history of Arunachal Himalaya is complex and has been influenced by several major tectonic events, including the 1950 M_w_8.6 Assam-Tibet earthquake. In this study, we explored the effect of dynamic stresses generated by teleseismic events on the triggering of seismicity in the region. We analyzed 34 large teleseismic events since 2010 and found triggering during six events. The change in seismicity was also confirmed by analysis with the STA/LTA method. The triggering in the region occurred in the form of earthquakes and tremors. The dynamic stress as low as 1 kPa was found capable of triggering. The back-azimuth angle does not play an important role in the triggering. The angle direction of incoming waves with respect to the fault ~ 60° and ~ 120° is the possible reason for triggering in the region. The triggering occurred in the Mishmi and Main Central Thrust regions. The largest triggered event, M_L_2.3, was triggered 7.5 h after the 2012 Indian Ocean earthquake of M_w_8.6. The region is tectonically very sensitive and tiny stresses are capable of triggering seismicity in Arunachal Pradesh.

## Introduction

The dynamic triggering of seismicity refers to the phenomena in which seismic events, such as earthquakes or tremors, may be triggered by the passage of seismic waves from other earthquakes^1–10^. Triggered events are generally identified in the form of earthquakes or non-volcanic tremors and are usually observed in areas where human activities occur^4,11,12^ and in active plate boundaries^13^. The phenomenon generally occurs due to the perturbation of large amplitude surface waves on a critically stressed region^14–18^. At distant locations, these waves have the potential to alter the stress and strain fields in the region, which are sometimes enough to increase seismicity. Many recent studies have shown that dynamic triggering of earthquakes could be a function of mainshock rupture direction^19,20^, amplitude and frequency^5,8,21,22^ and direction of the incoming wave with respect to the fault orientation^15,23^. New methodologies and approaches are being developed to understand the elements that drive dynamic triggering and to improve earthquake forecasting and vulnerability analysis in earthquake-prone areas^13^.

The study of dynamic triggering plays an important role to understand seismic hazard in Arunachal Pradesh, an area in Northeastern India located in the seismically active Himalayan zone. The region is very vulnerable to seismic activity due to its location where the Indian and Eurasian tectonic plates collide. The area is prone to earthquakes because of the complex structure of faults and fractures. The region is also well-known for the complexity of its geological characteristics^24^. The biggest recorded earthquake in this region was M_w_8.6 in 1950, which severely damaged Arunachal Pradesh and the neighbouring areas of Northeastern India^25^.

Earlier studies were conducted on dynamic triggering in the south-central Tibet, the southwest China region of the Himalayas^20,26^ and the central Himalayas^27^. The Sumatra, 2004, M_w_9.1 and Nias earthquake, 2005, M_w_8.6 triggered a local earthquake of M_L_ ≤ 4 in the Gaize region, north of the Banging-Nujiang Suture Zone separating the Lhasa and Qiangtang Terranes, south-central Tibet^26^. The seismicity increases during 50 h and a few hours after the Sumatra, 2004, and Nias, 2005 earthquakes in south-central Tibet^26^. The frequency of earthquakes in the Kumaon-Garhwal, central Himalayan area, increased six-fold within 12 hours after the arrival of teleseismic waves of the 2007 M_w_8.5 Sumatra earthquake^27^. The Yunnan and Tengchong volcanic regions in southwest China experienced an increase in seismic activity following the 2004 M_w_9.1 Sumatra earthquake^20^. The highest local magnitude earthquake, M_L_4.7, occurs in the Tengchong volcanic region. The seismic activity rate remained uniform after the 2012 M_w_8.6 Indian Ocean earthquake in Yunnan and the Tengchong region. After a few days, the frequency of earthquakes increases, indicating a probable delay in triggering in the region after the 2012 Indian Ocean earthquake^20^. The above studies analyzed only one or two teleseismic earthquakes in different parts of the Himalaya, excluding Arunachal Pradesh. Hence, we are carrying out a systematic study of dynamic triggering in the Arunachal Himalayas. In this study, we attempted to identify the triggering in Arunachal Pradesh and explore the causes of the triggering.

## Study region

**Figure 1:**
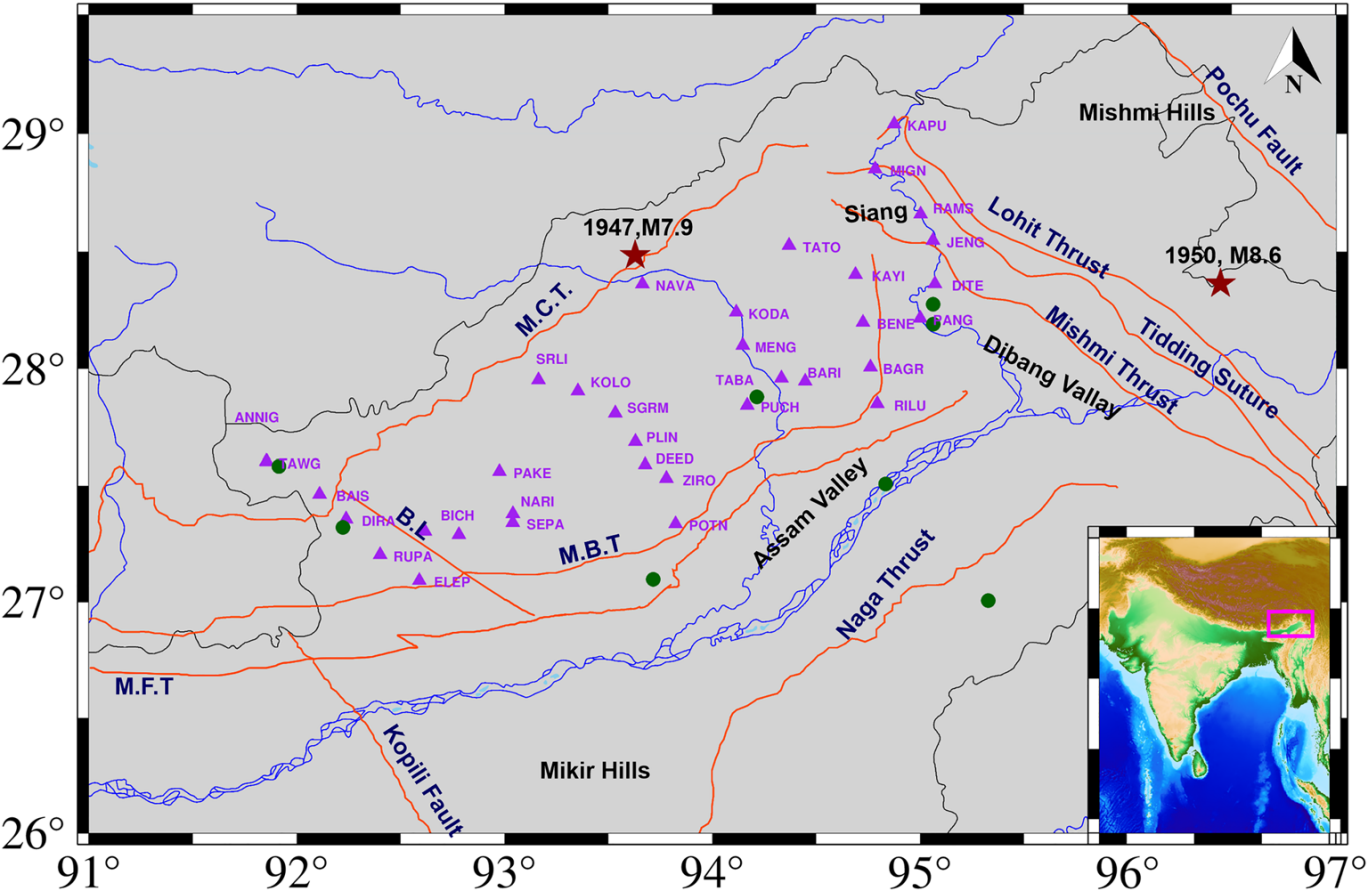
Tectonic map of Arunachal Himalaya and seismic stations (purple triangles), where green solid circle represents the hot spring. MCT: Main Central Thrust, MBT: Main Boundary Thrust, MFT: Main Frontal Thrust, BL: Bomdilla Lineament. The Seismotectonic data used for plotting the faults (solid orange line) and hot spring, Geological Survey of India, Government of India, Kolkata, India, last access on 09 October 2023, https://bhukosh.gsi.gov.in/Bhukosh/Public. The figure is made using GMT version 6.3.0.

Arunachal Himalaya covers an area between latitudes 26° 40′ N to 29° 25′ N and longitudes 91° 35′ to 97° 25′ E in the northeastern Himalayas. The northeastern Himalayas and its surrounding region are dominated by N–S oriented compressive tectonic stresses^28^. The region has two major’s tectonic units viz. the Main Central Thrust (MCT) and the Main Boundary Thrust (MBT). The seismicity of the MBT bordering the lesser Himalayas and Tertiary layers is well-recognized, and numerous significant earthquakes have occurred along the boundary in the past^28^. The MCT is a significant intercontinental shear zone that separates the crystalline higher Himalayas in the north from the lesser Himalayas in the south. Other significant faults in the region comprise the Kopili Fault, Lohit Thrust, and Mishmi Thrus^29^ (Fig. 1). Approximately 32 earthquakes of magnitudes ≥ 6 have occurred in 121 years (1900–2021) (ANSS database), which shows that the region is tectonically active and critically stressed. The region is also characterized by many hot springs and geysers^24^. One of the most prominent recent earthquakes was the M_w_6.4 earthquake on January 4, 2005, with its epicenter near the India-Bhutan border. An event of M_w_5.5 impacted the region near Assam-Arunachal Himalaya on April 24, 2019. The Arunachal Himalaya can be divided into the western and eastern regions by the Siang Window^30–32^.

## Data and method

We selected 34 events that generated theoretical dynamic stresses ≥ 1 kPa at RUPA station. The theoretical dynamic stress in the area is computed^33^ using

$${\text{V}} \approx {2}\pi {\text{A}}_{{{2}0}} /{\text{T}}$$

Here T = 20 s and the value of A_20_ are find out using the surface wave magnitude relation $log_{10} A_{20}$ = $M_{s}$ − 1.66 ${ }log_{10}$ Δ − 2, Where A_20_ is in micrometers and Δ in degree^33^. The observed dynamic stresses are presented in Table 1.

**Table 1:** The events in Arunachal Pradesh that were examined for dynamic triggering.

Date, time	Lat	Long	Mag	Depth	Place	BAZ range	Mean distance (km)	Peak Dynamic Stress (kPa)
2010-02-27, 06:34:11.35	− 36.122	− 72.898	8.8	22.9	36 km WNW of Quirihue, Chile	230–232	18,310	4.1
2010-03-30, 16:54:46.73	13.667	92.831	6.6	34	The Andaman Islands, India region	178–191	1550	1.6
2010-01-13, 23:49:38.33	33.165	96.548	6.9	17	Southern Qinghai, China	7–30	695	1.3
2010-04-14, 01:25:15.58	33.195	96.449	6.1	7.6	Southern Qinghai, China	6–29	694	14.8
2010-04-16, 01:45:15.74	54.485	161.039	5.7	34.3	Alaska Peninsula	34–35	8572	1.2
2010-05-09, 05:59:41.62	3.748	96.018	7.2	38	Northern Sumatra, Indonesia	170–179	2659	2.6
2010-12-21, 17:19:40.66	26.901	143.698	7.4	14	Bonin Islands, Japan region	78–80	4858	1.4
2010-04-06, 22:15:01.58	2.383	97.048	7.8	31	Northern Sumatra, Indonesia	167–169	2802	9.4
2010-06-12, 19:26:50.46	7.881	91.936	7.5	35	Nicobar Islands, India region	181–183	630	7.3
2010-10-25, 14:42:22.46	− 3.487	100.082	7.8	20.1	Kepulauan Mentawai region, Indonesia	165–172	3518	6.4
2011-01-18, 20:23:23.48	28.777	63.951	7.2	68	Southwestern Pakistan	277	3109	2.3
2011-03-09, 02:45:20.33	38.435	142.842	7.3	32	Near the east coast of Honshu, Japan	61–63	4669	1.9
2011-10-23, 10:41:23.25	38.721	43.508	7.1	18	Eastern Turkey	297–298	4792	1.25
2011-03-11,05:46:24.12	38.297	142.373	9.1	29	Tohoku, Japan	61–63	4630	76.8
2012-01-10, 18:36:59.08	2.433	93.21	7.2	19	off the west coast of northern Sumatra	177–184	2812	2.43
2012-04-11, 08:38:36.72	2.327	93.063	8.6	20	off the west coast of northern Sumatra	177–184	2836	60.8
2012-04-11, 10:13:10.85	0.802	92.463	8.2	25.1	off the west coast of northern Sumatra	179–186	3016	21.9
2012-08-31, 12:47:33.38	10.811	126.638	7.6	28	Philippine Islands region	110–114	3908	3.2
2012-12-07, 08:18:23.13	37.89	143.949	7.3	31	off the east coast of Honshu, Japan	62–64	4768	1.2
2013-02-06,01:12:25.83	− 10.799	165.114	8	24	76 km W of Lata, Solomon Islands	107–109	8781	2.2
2013-06-05, 04:47:26.24	− 11.401	166.299	6.1	39	91 km SSE of Lata, Solomon Islands	107–109	9304	1.6
2013-10-15, 00:12:32	9.8796	124.116	7.1	19.04	4 km SE of Sagbayan, Philippines	113–118	3725	1.1
2013-04-16, 10:44:20:18	28.033	61.996	7.7	80	83 km E of Khash, Iran	276–279	3117	6.7
2014-02-12, 09:19:49.06	35.9053	82.586	6.9	10	272 km ESE of Hotan, China	307–318	1386	4
2014-04-01, 23:46:47.26	− 19.609	− 70.769	8.2	25	94 km NW of Iquique, Chile	292–302	18,213	1.06
2015-04-25, 06:11:25.95	28.2305	84.7314	7.8	8.22	36 km E of Khudi, Nepal	268–279	956	210.5
2015-05-12, 07:05:19.73	27.8087	86.0655	7.3	15	19 km SE of Kodari, Nepal	264–277	768	180.6
2015-09-16, 22:54:32.86	− 31.572	− 71.674	8.3	22.44	48 km W of Illapel, Chile	248–253	18,567	1.3
2015-12-07,07:50:05:95	38.2107	72.7797	7.2	22	104 km W of Murghob, Tajikistan	302–308	2285	3.5
2016-03-02, 12:49:48.11	− 4.9521	94.3299	7.8	24	Southwest of Sumatra, Indonesia	175–182	3648	6.2
2016-08-24, 10:34:54.58	20.9228	94.569	6.8	82	26 km W of Chauk, Burma	163–190	735	62.6
2016-12-08, 17:38:46.28	− 10.681	161.327	7.8	40	69 km WSW of Kirakira, Solomon Islands	110–111	8378	1.5
2016-12-17, 10:51:10.5	− 4.5049	153.521	7.9	94.54	54 km E of Taron, Papua New Guinea	109–110	7282	2.3
2018-06-06, 13:15:52:49	58.87	− 166.73	8.86	4.96	Northern Osaka earthquake, Japan	32–33	7821	17.7

The events induced dynamic stresses of ≥ 1 kPa at the RUPA seismic station. For each station mean distance of the station from the event, range of backazimuth at all analyzed stations and the dynamic stress of each event were calculated. The regions peak dynamic stress may be calculated^14^ using the peak ground velocity as σ = $\frac{{\mu \left( {PGV} \right)}}{v}$, where σ, μ, PGV and* v* are dynamic stress, shear rigidity, Peak ground velocity, and surface wave phase velocity, respectively with an assumption of phase velocity of 3.5 km/s and shear rigidity of 35 GPa.

**Figure 2:**
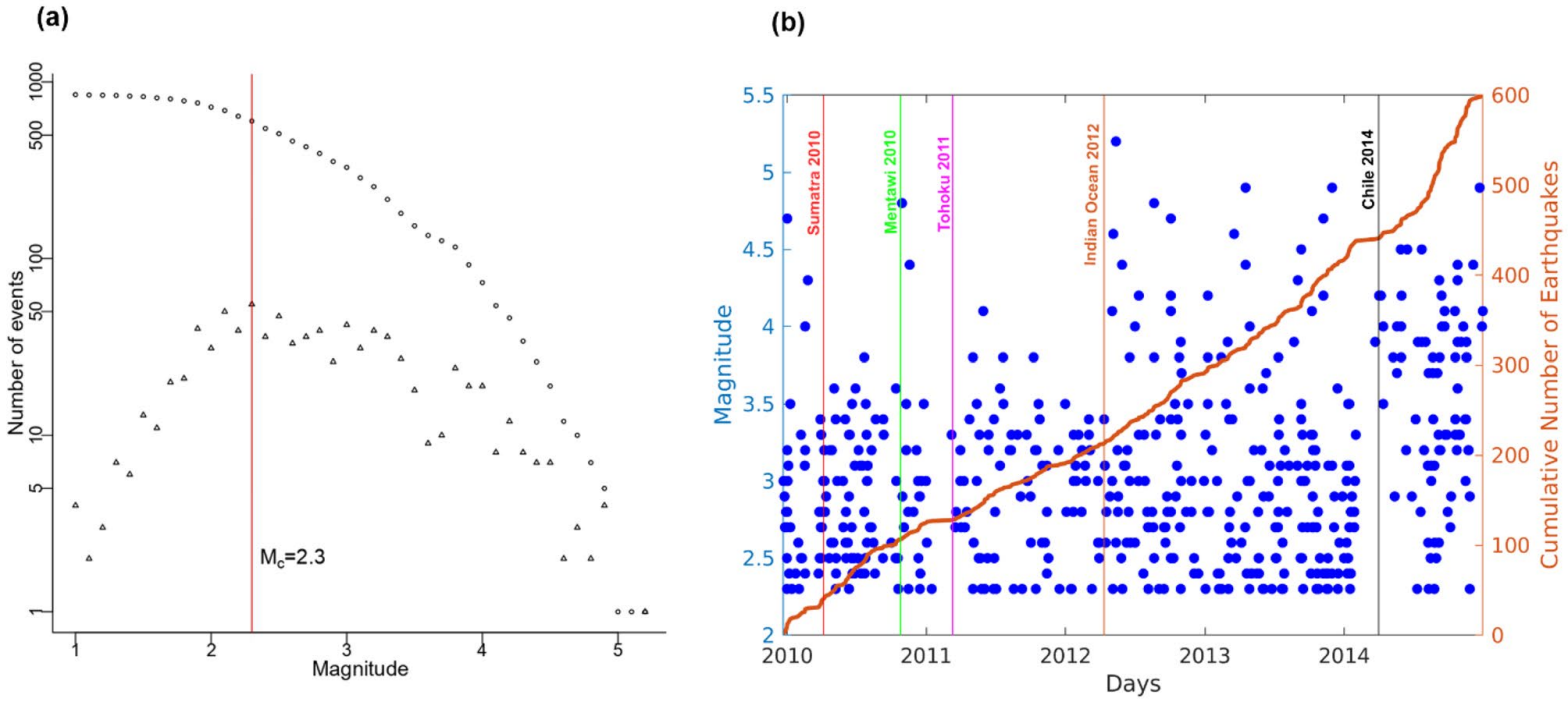
(**a**) Plot of the log number of events shown in empty triangles and log of a cumulative number of events shown in empty circles versus magnitude. The red vertical line corresponds to the magnitude of the completeness of 2.3. (**b**) A temporal plot of the seismicity where variously coloured vertical lines depict the occurrence of trigger events. The cumulative number of events (solid orange line) does not indicate any substantial rise after the main shock arrival.

The CSIR-NGRI, Hyderabad has been operating a seismic network of 34 broadband stations in the Arunachal Himalayas since 2010 (Fig. 1, Supplementary Table ST1). We examine the local catalog from years 2010 to 2018 comprising 848 events in Arunachal Pradesh. The local catalog's magnitude of completeness (M_c_) was determined by using the maximum curvature method and found to be 2.3 (Fig. 2a). The M_c_ is an essential element in figuring out data completeness^34,35^. The cumulative plot shows no important change in seismicity following all significant events (Fig. 2b). The catalog data typically lacks the smaller events that may be detected by waveform analysis; hence the waveform data give more information on the dynamic triggering than the catalog data^3,8,9,17,36^.

After catalog analysis we analyzed the waveform data from this network to study the triggering. The waveform data of 24 h duration were analyzed to find the signature of triggering in the region. The instrument corrections were applied to the waveform data. The instrumental corrected components of N–S and E–W were rotated to a great circle path to obtain the transverse and radial components. The waveform data was high passed at 5 Hz to obtain the signature of local events since remotely triggered events generally have greater frequencies than the teleseismic event^2^. We generated the spectrogram of the waveform data to identify the triggered events using a short window Fourier transform. The waveform is analyzed manually to identify the triggered tremors or earthquakes. The statistical significance of the change in seismicity is computed using the β value. A longer duration of ~ 6 h of background seismicity is essential to estimate the β value, which can be estimated as^37,38^.

$$\upbeta = \frac{{{\text{N}}_{{\text{a}}} - {\text{N}}\left( {\frac{{{\text{T}}_{{\text{a}}} }}{{\text{T}}}} \right)}}{{\sqrt {\left( {{\text{N}}\left( {\frac{{{\text{T}}_{{\text{a}}} }}{{\text{T}}}} \right)} \right)\left( {1 - \frac{{{\text{T}}_{{\text{a}}} }}{{\text{T}}}} \right)} }}$$

where ${\text{N}}_{{\text{a}}}$ and N are the number of events after the main shock in the targeted triggered window and the total number of events during the background and triggered window, respectively, T and ${\text{T}}_{{\text{a}}}$ are the total time window and duration of the triggered window respectively. A β value of 2 implies an increase in the seismicity rate, whereas a β value of -2 suggests a significant drop in the seismicity rate^13^.

## Results

At the outset, during the examination of the waveform data of mainshocks, we used spectrogram and visual analysis to identify high-frequency microearthquakes and tremors during the large-amplitude surface waves of 34 teleseismic events and found triggering during six teleseismic events, (1) April 6, 2010, M_w_7.8 Sumatra, (2) October 25, 2010, M_w_7.8 Mentawai, (3) March 11, 2011, M_w_ 9.1 Tohoku-Oki, (4) April 11, 2012, M_w_8.6 Indian Ocean (5) April 11, 2012, M_w_8.2 Indian Ocean aftershock, (6) April 1, 2014, M_w_8.2 Iquique earthquake.

The Banyak Islands, Sumatra earthquake occurred on April 6, 2010, at 22:15 UTC with a magnitude of M_w_7.8 and thrust faulting mechanism at the plate boundary between the Australia-India and Sunda plates^39^. The peak dynamic stresses vary from 1.79 to 3.09 kPa with an average of 2.21 kPa. The regions peak dynamic stress calculated using the peak ground velocity as σ = (μ(PGV))/v, where σ, μ, PGV and v are dynamic stress, shear rigidity, Peak ground velocity, and surface wave phase velocity, respectively^14^ with an assumption of phase velocity of 3.5 km/s and shear rigidity of 35 GPa.

**Figure 3:**
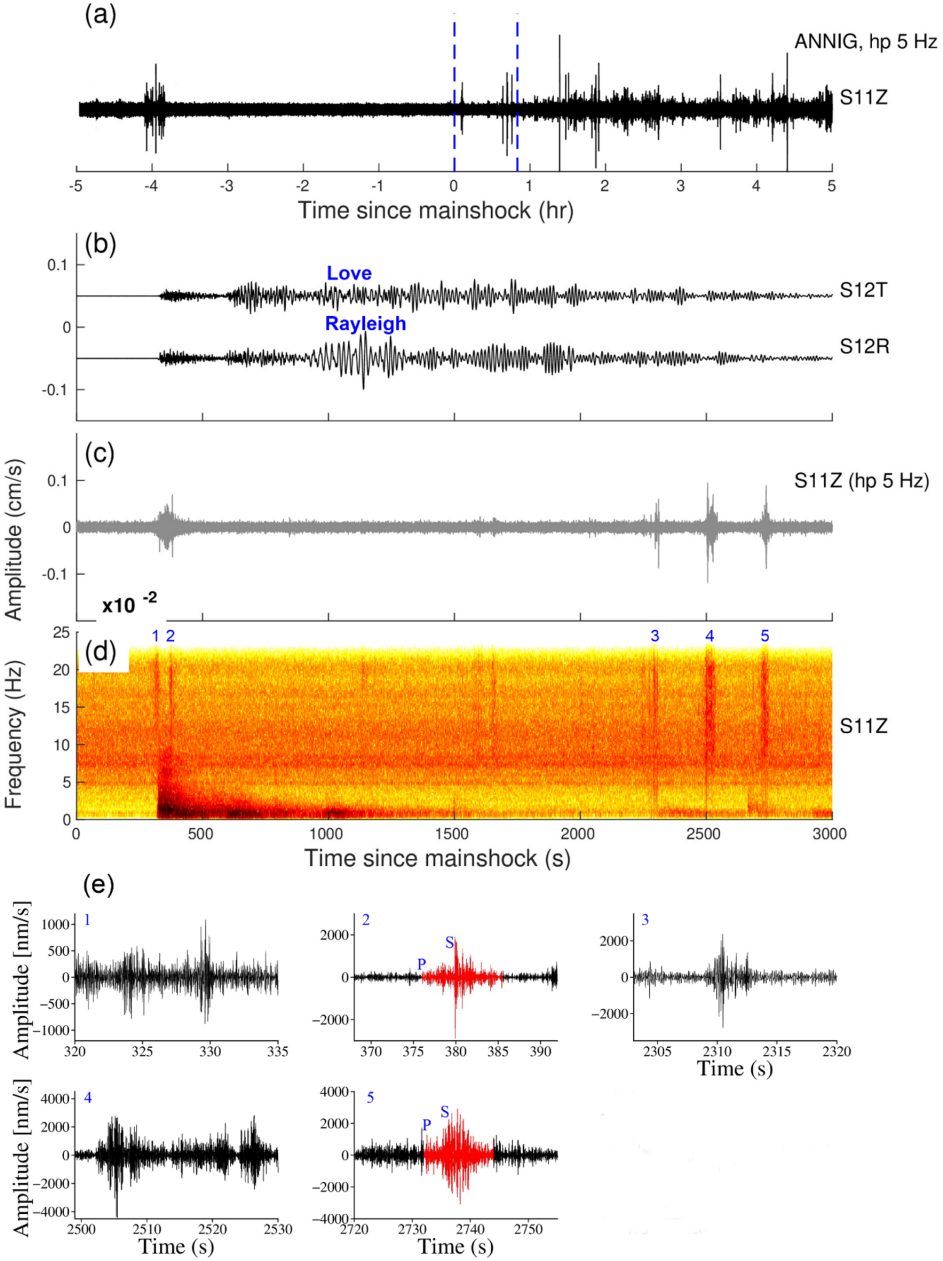
(**a**) Vertical components of the 5 Hz band passed waveform at ANNIG station during the Sumatra earthquake, April 6, 2010, M_w_7.8, (**b**), (**c**) and (**d**) are Zooming portion of blue vertical line in (**a**), (**b**) Transverse and Radial component, (**c**) Vertical component with a 5 Hz high pass filter, (**d**) Vertical component spectrogram, (**e**) zoom in the portion of the number marked on the spectrogram. Red and black colour waveforms represent (high pass 5 Hz) the microearthquakes and tremors in (**e**). (**b**) and (**c**) Follow the same time scale of (**d**).

The triggering is found at the ANNIG station which is 6 km away from the hot spring (Fig. 1) and peak dynamic stress was 3.09 kPa. The triggering phenomenon has been observed to occur within 300–400 s in the body wave phase and between 2000 and 3000 s in the surface wave passage (Fig. 3). The β value for the ANNIG station was 4.5 (Supplementary Fig. S1) which indicates a statistically significant increase in the seismicity.

**Figure 4:**
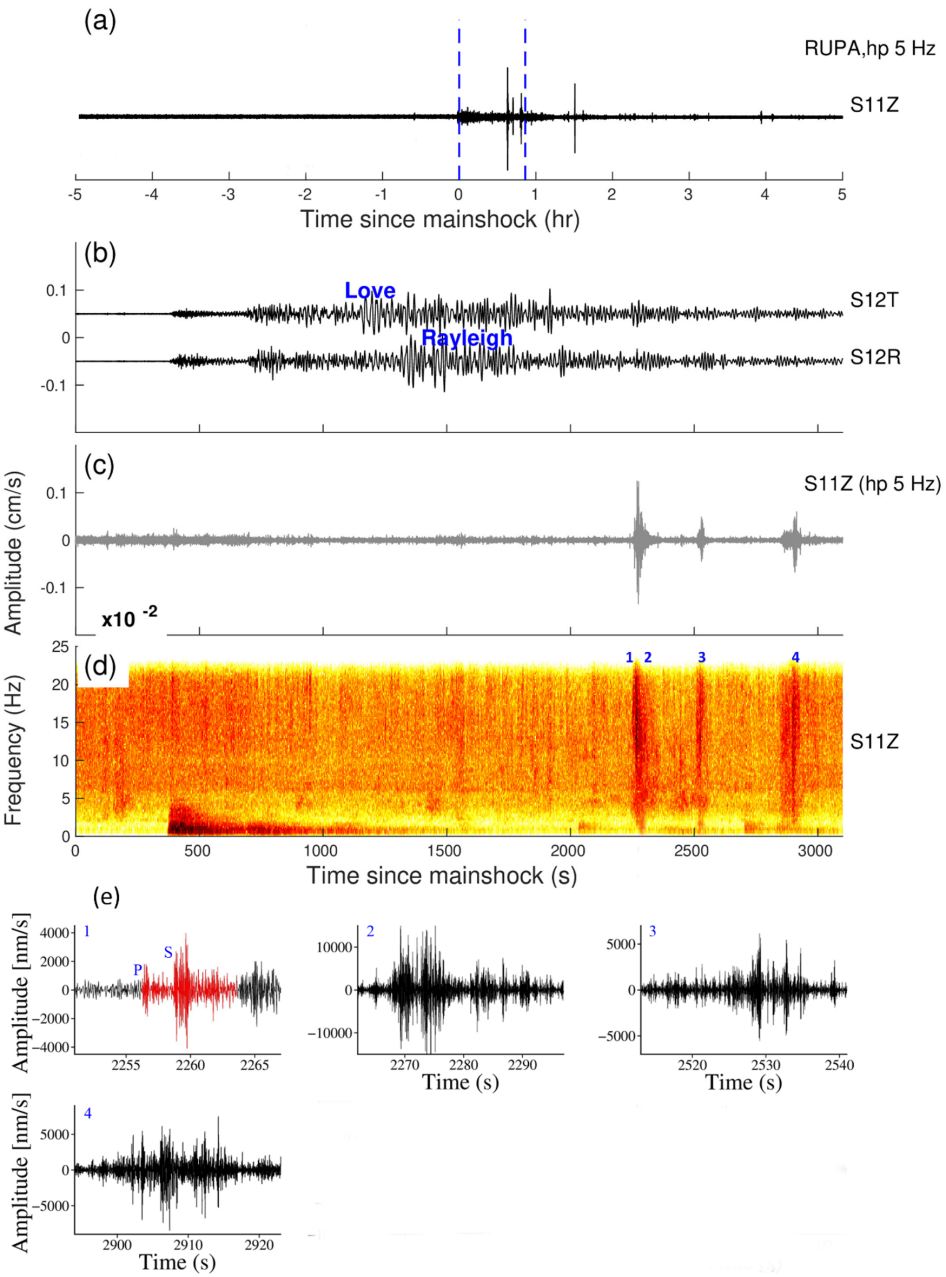
(**a**) Vertical components of the 5 Hz band passed waveform at RUPA station during the Mentawai earthquake, October 25, 2010, M_w_7.8, (**b**), (**c**) and (**d**) are Zooming portion of blue vertical line in (**a**), (**b**) Transverse and Radial component, (**c**) Vertical component with a 5 Hz high pass filter, (**d**) Vertical component spectrogram, **(e)** zoom in the portion of the number marked on the spectrogram. Red and black colour waveforms represent (high pass 5 Hz) the triggered micro earthquakes and tremors in (**e**). (**b**) and (**c**) Follow the same time scale of (**d**).

The Mentawai earthquake on the western coast of Sumatra occurred at 14:42 UTC on October 25, 2010, M_w_7.8 with a thrust faulting mechanism. It generated a large localized tsunami that hit the Mentawai Islands^40^. The Mentawai earthquake was recorded in 10 Indian stations and we find the triggering at RUPA station (Fig. 4). The triggered events are observed between 2200 and 3000 s during the surface waves and we recorded one microearthquake and three tremors during that duration.

**Figure 5:**
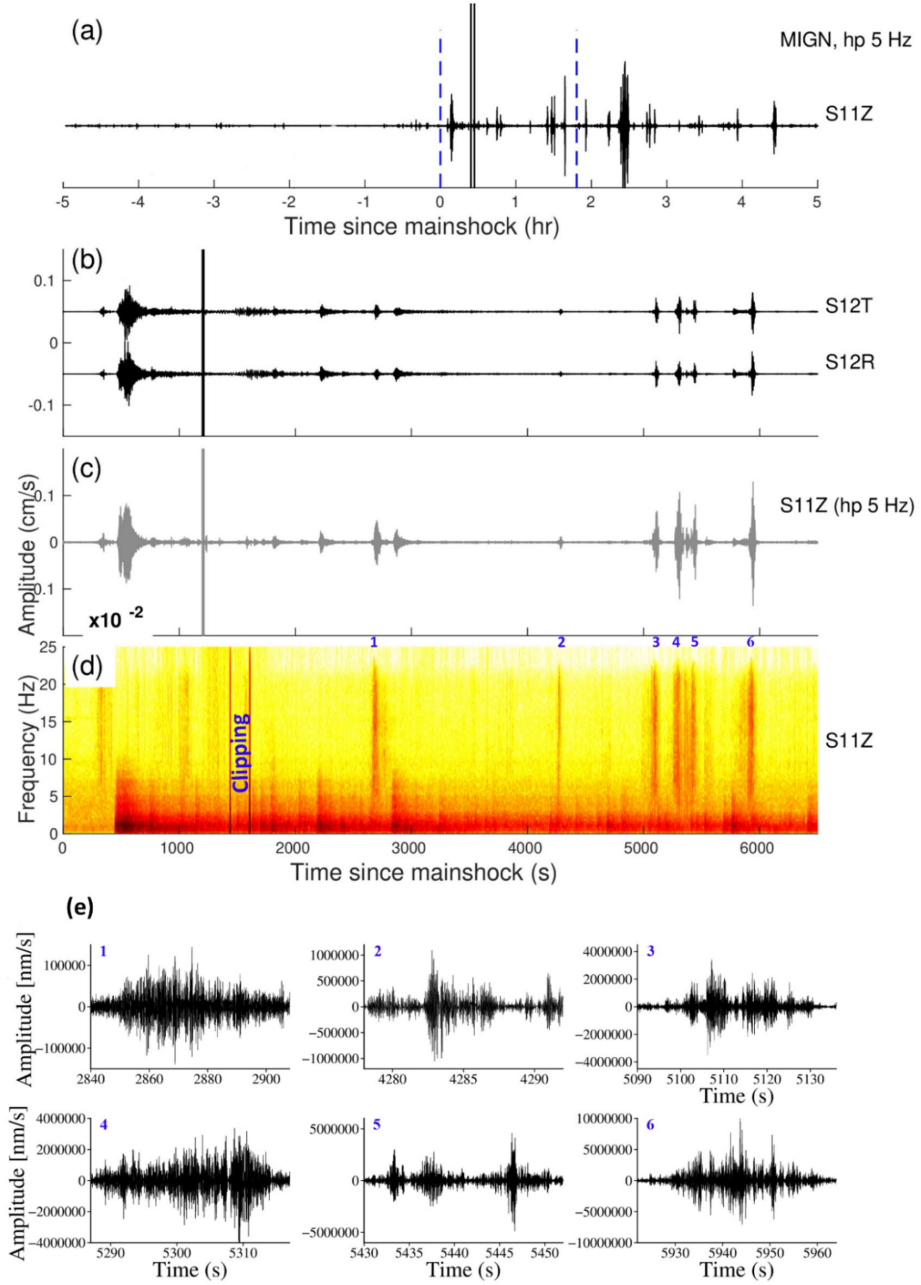
(**a**) Vertical components of the 5 Hz band passed waveform at MIGN station during the Tōhoku earthquake, March 11, 2011, M_w_9.1, (**b**), (**c**) and (**d**) are Zooming portion of the blue vertical line in (**a**), (**b**) Transverse and Radial component, (**c**) Vertical component with a 5 Hz high pass filter, (**d**) Vertical component spectrogram, (**e**) zoom in the portion of the number marked on the spectrogram and black colour waveform represent (high pass 5 Hz) triggered tremors. (**b**) and (**c**) Follow the same time scale of (**d**).

**Figure 6:**
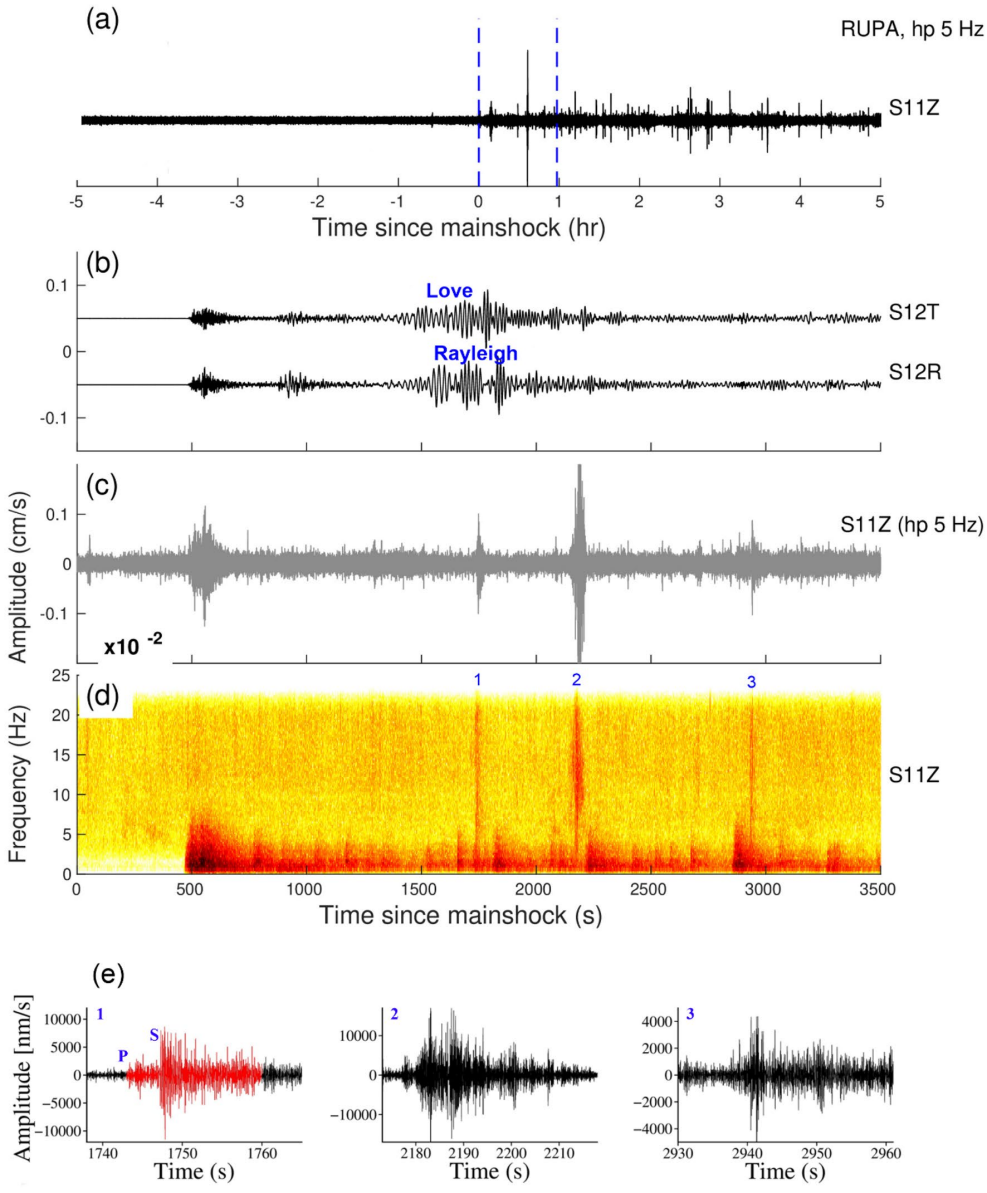
(**a**) Vertical components of the 5 Hz band passed waveform at RUPA station during the Tōhoku earthquake, March 11, 2011, M_w_9.1, (**b**), (**c**) and (**d**) are Zooming portion of blue vertical line in (**a**), (**b**) Transverse and Radial component, (**c**) Vertical component with a 5 Hz high pass filter, (**d**) Vertical component spectrogram, (**e**) zoom in the portion of the number marked on the spectrogram. Red and black colour waveforms represent (high pass 5 Hz) the triggered microearthquakes and tremors in (**e**). (**b**) and (**c**) Follow the same time scale of (**d**).

The Tohoku earthquake occurred on March 11, 2011, M_w_9.1 at 05:46:24 UTC, near the east coast of Honshu, Japan with a thrust faulting mechanism on the Pacific-North America sub-duction zone. The earthquake generated peak dynamic stresses of 12.4–16 kPa at the vertical component, averaging 13.97 kPa. The triggering is detected at the MIGN (Fig. 5) and RUPA (Fig. 6) stations. The triggering at the MIGN and RUPA stations was initiated during the surface waves. At the MIGN station, we observed the triggered tremors and at the RUPA station during the time period of 1700–3000 s, we observed one microearthquake and two tremors. Both the stations show evidence of delayed triggering (Figs. 5, 6). The β value at RUPA and MIGN station is 2.5 and 7.09, respectively.

**Figure 7:**
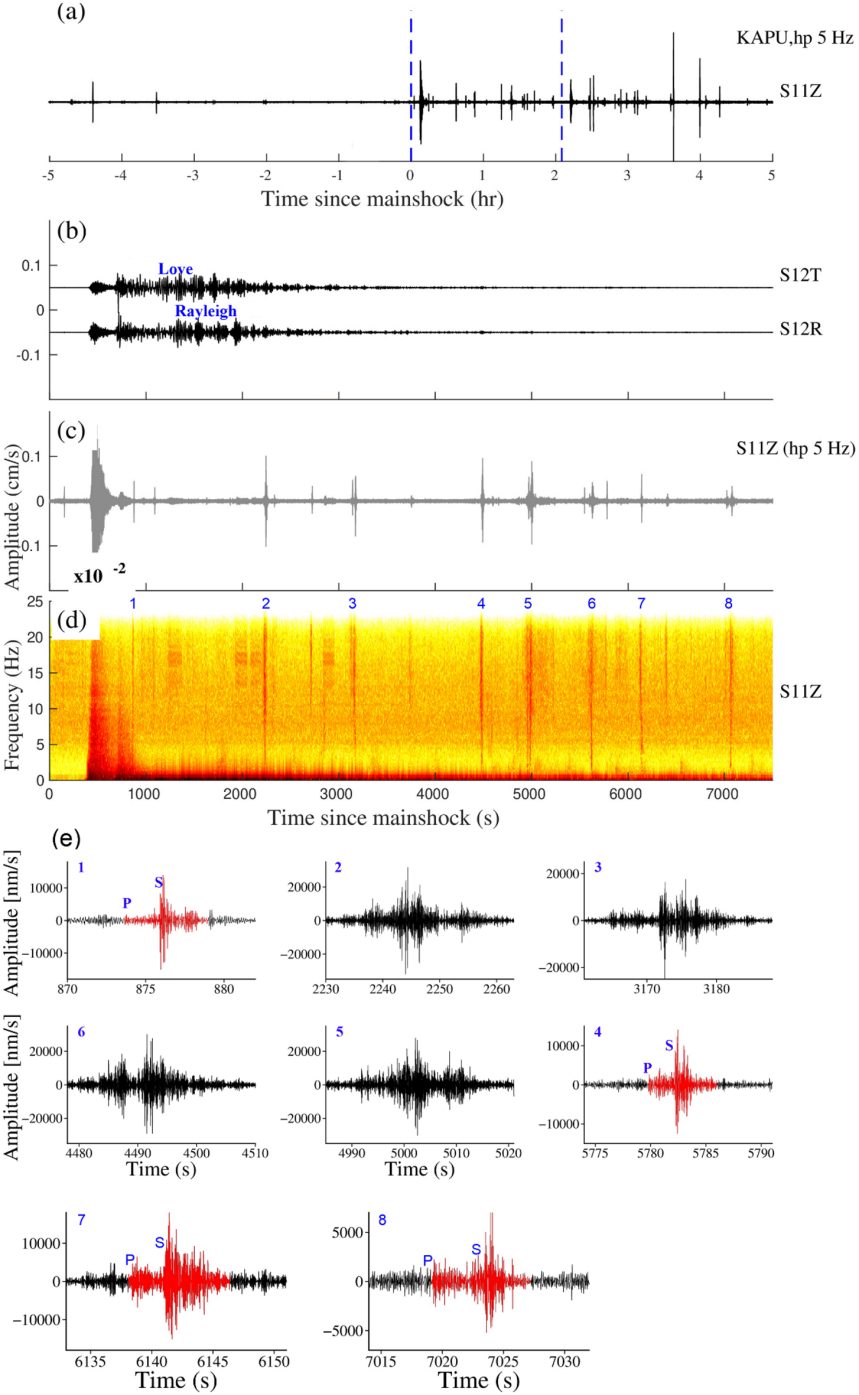
(**a**) Vertical components of the 5 Hz band passed waveform at KAPU station during the Indian Ocean earthquake, April 11, 2012, M_w_8.6, (**b**), (**c**) and (**d**) are Zooming portion of blue vertical line in (**a**), (**b**) Transverse and Radial component, (**c**) Vertical component with a 5 Hz high pass filter, (**d**) Vertical component spectrogram, (**e**) zoom in the portion of the number marked on the spectrogram. Red and black colour waveforms represent (high pass 5 Hz) the triggered microearthquakes and tremors in (**e**). (**b**) and (**c**) Follow the same time scale of (**d**).

**Figure 8:**
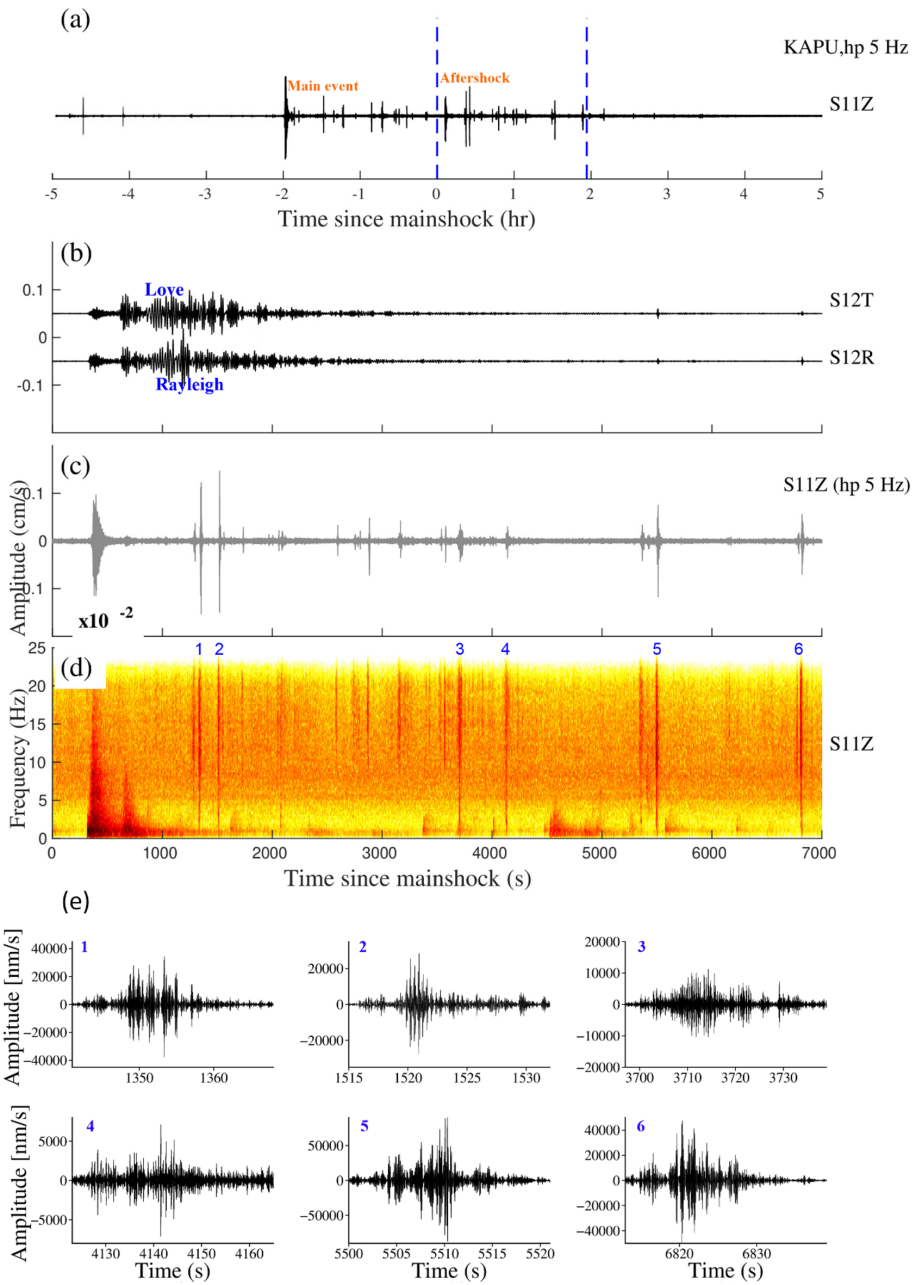
(**a**) Vertical components of the 5 Hz band passed waveform at KAPU station during the Indian Ocean earthquake (Aftershock), April 11, 2012, M_w_8.2, (**b**), (**c**) and (**d**) are Zooming portion of blue vertical line in (**a**), (**b**) Transverse and Radial component, (**c**) Vertical component with a 5 Hz high pass filter, (**d**) Vertical component spectrogram, (**e**) zoom in the portion of the number marked on the spectrogram and black colour waveform represent (high pass 5 Hz) triggered tremors. (**b**) and (**c**) Follow the same time scale of (**d**).

The M_w_8.6 earthquake in the eastern Indian Ocean on April 11, 2012, is the largest strike-slip event on record. This earthquake caused a sudden and significant increase in seismic activity worldwide^41^. The events were detected at the KAPU station in the form of instantaneous and delayed triggering (Fig. 7). We observed triggering in the form of microearthquakes and tremors. The KAPU station was also triggered during its largest aftershock of M_w_8.2 (Fig. 8). The Indian Ocean earthquake and its largest aftershock generate β value 2.69 and 3.78 at the KAPU station. Specifically, two earthquakes with magnitudes of M_L_1.8 and 2.3 were recorded at around 4.6 and 7.5 h, respectively after Indian Ocean main shock.

**Figure 9:**
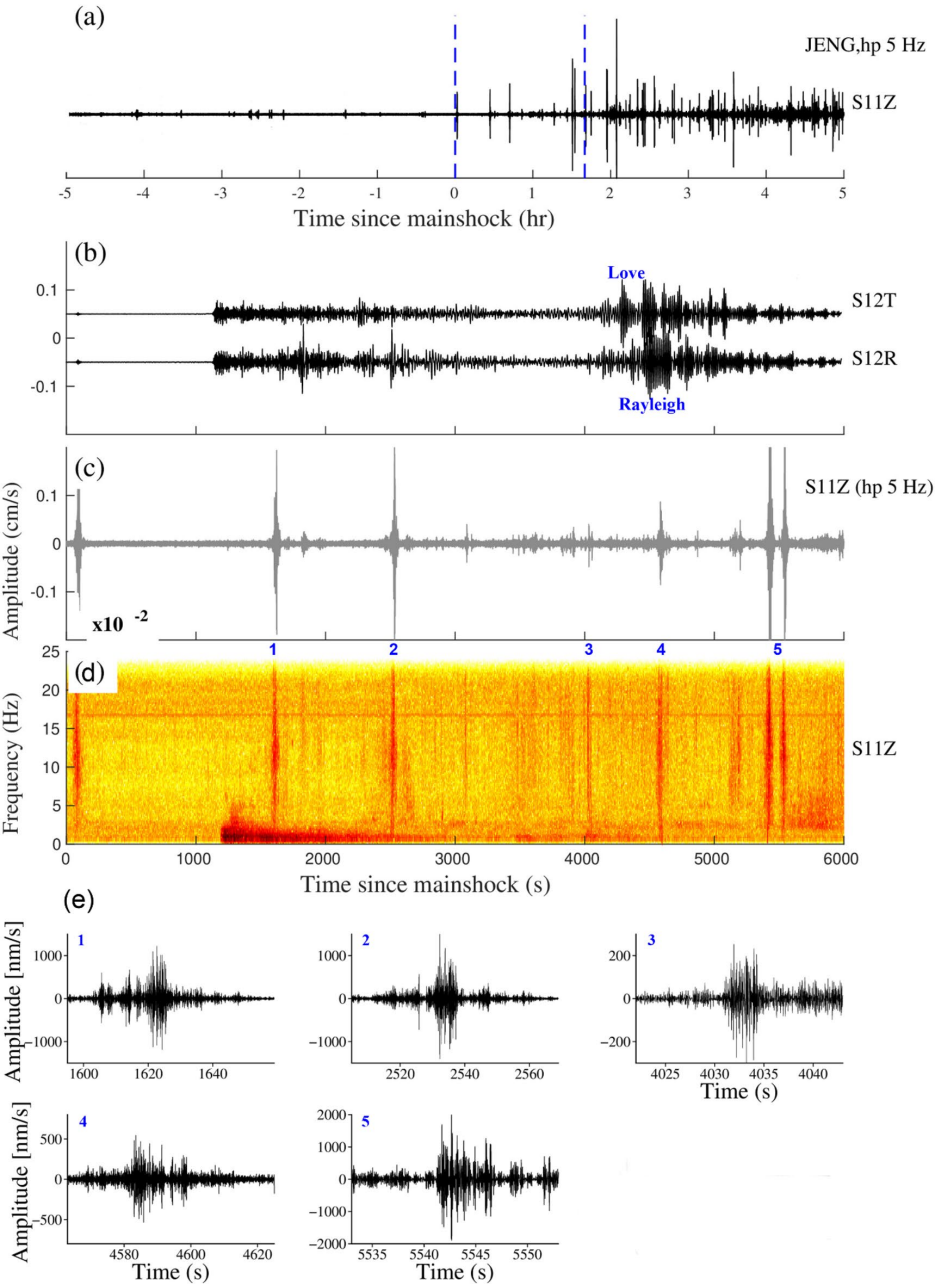
(**a**) Vertical components of the 5 Hz band passed waveform at JENG station during the Iquique earthquake, April 1, 2014, M_w_8.2, (**b**), (**c**) and (**d**) are Zooming portion of the blue vertical line in (**a**), (**b**) Transverse and Radial component, (**c**) Vertical component with a 5 Hz high pass filter, (**d**) Vertical component spectrogram, (**e**) zoom in the portion of the number marked on the spectrogram and black colour waveform represent (high pass 5 Hz) triggered tremors. (**b**) and (**c**) Follow the same time scale of (**d**).

The Iquique earthquake occurred on April 1, 2014, M_w_8.2 at 23:46 UTC, off the coast of Chile. The earthquake occurred due to shallow depth thrust faulting between the Nazca and South American plates, where the Nazca plate is subducting at a rate of 65 mm/year^42^. The earthquake was recorded at 20 stations. The Iquique earthquake was triggered at stations JENG (Fig. 9), PANG, and KAPU (Supplementary Fig. S2, S3). At all 3 stations, we observed the triggering in the form of tremors. All the triggered stations are near the Mishmi thrust and Lohit thrust which are active faults^29,30^. The β value of triggered stations is > 2, which indicates a statistically significant increase in the seismicity. The remaining stations do not show any sign of triggering except the TAWG station which possibly showed triggered tremors (Supplementary Fig. S4).

**Figure 10:**
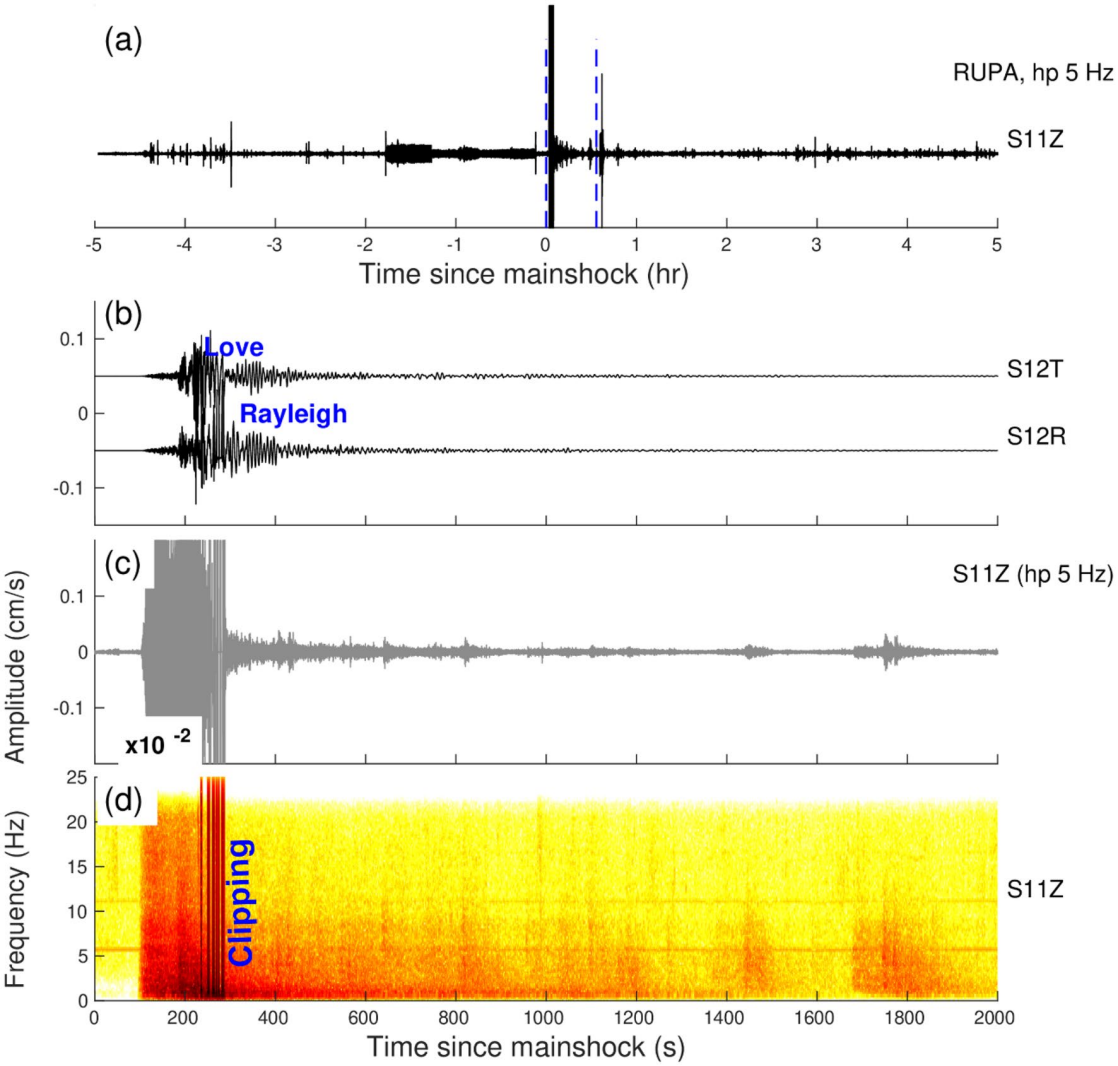
(**a**) Vertical components of the 5 Hz band passed waveform at RUPA station during the Nepal earthquake, April 25, 2015, M_w_7.8, (**b**) Transverse and Radial component, (**c**) Vertical component with a bandpass of 5 Hz, (**d**) Spectrogram of the vertical component.

The 2015 M_w_7.9 Nepal earthquake and its aftershock of M_w_7.3 generated dynamic stress of 201.5 and 180.6 kPa respectively. Despite having high dynamic stress, the Nepal earthquake was unable to trigger any seismicity in the study region (Fig. 10). Similarly, the earthquake with a magnitude of 6.8 occurred in Myanmar on August 24, 2016, with a peak dynamic stress of 62.6 kPa in the region, but unable to trigger events. The criticality of a region may be one of the criteria for triggering the seismicity in the region.

We do not find any triggering evidence on other stations for any events. We plotted the log envelope of non-triggering stations for the six events (Supplementary Fig. S6 to S10). We visually inspected all waveforms but could not find any sign of triggering at the rest of the stations.

## Discussion

In the study, we systematically analyzed the dynamic triggering in the Arunachal Himalayas following large and shallow teleseismic earthquakes. Numerous studies provided evidence of dynamic triggering along major plate boundaries or volcanic/geothermal areas^8,14,43^ and stable intraplate locations^19,22,23,44,45^. Examining dynamic triggering is a valuable tool for recognizing the appearance of earthquakes or tremors within a particular area due to minor stress perturbations.

Arunachal Himalaya is situated in a region prone to seismic activity owing to its proximity to the eastern segment of the Indian Plate boundary, where it comes into contact with the Eurasian Plate. This area is situated within the wider seismic zone of the Himalayan region and exhibits susceptibility to seismic activity. Earlier cases of dynamic triggering in the Himalayan region were found in south-central Tibet following the 2004 M_w_9.1 Sumatra & 2005 M_w_8.6 Nias earthquakes^26^, central Himalaya during the 2007 M_w_8.5 Sumatra earthquake^27^, and the southwest China during the 2004 M_w_9.1 Sumatra earthquake & 2012 M_w_8.6 Indian Ocean earthquake^20^.

We examined 34 teleseismic events and identified the triggered event at six stations (ANNIG, RUPA, MIGN, KAPU, PANG, and JENG) following the six remote main events, namely, 2010 M_w_7.8 Sumatra, 2010 M_w_7.8 Mentawai, 2011 M_w_ 9.1 Tohoku-Oki, 2012 M_w_8.6 Indian Ocean, 2012 M_w_8.2 Indian Ocean aftershock, and 2014 M_w_8.2 Iquique earthquake. There are two basic hypotheses for understanding dynamic triggering: one based on the Coulomb failure criteria and the other linked with fluid movement or aseismic creep activation^13^. The delayed triggering is caused by fluid movement or aseismic creep, whereas the instantaneous triggering is caused by coulomb failure. Since we found both instantaneous and delayed triggering in our research, we believe that both models are responsible for triggering. It is interesting to note that previously a low-velocity zone (V_s_ = 3.38 km/s) was found at a depth of 20–30 km by joint inversion indicating a weak mid-crustal zone^46^. Microearthquakes are generally found to occur at shallower depths ≤ 15 km in the region^47^. There are potential factors that may contribute to the occurrence of triggering in Arunachal Himalaya, with one of them being the presence of hot springs (Fig. 1). Figure 1 illustrates the proximity of ANNIG, TAWG, JENG, and PANG to the hot springs, which are seen as a potential factor in the initiation of triggering. Dynamic stresses cause fluids to migrate along fault pathways, altering the stress distribution within the Earth's crust; the fluid migration and the associated changes in stress can temporarily stabilize fault segments by increasing confining pressure and inhibiting immediate slip along the fault^48^. The triggered stations KAPU, JENG, and PANG are around 80–120 km and 20–80 km from the Mishmi and Lohit thrusts respectively (Fig. 1), both of which are seismically active^49^. The most recent strong earthquake in the Mishmi Thrust^50^ was M_w_6.5 in 2000. The KAPU station was also triggered following the 2012 Indian Ocean and its aftershock.

**Figure 11:**
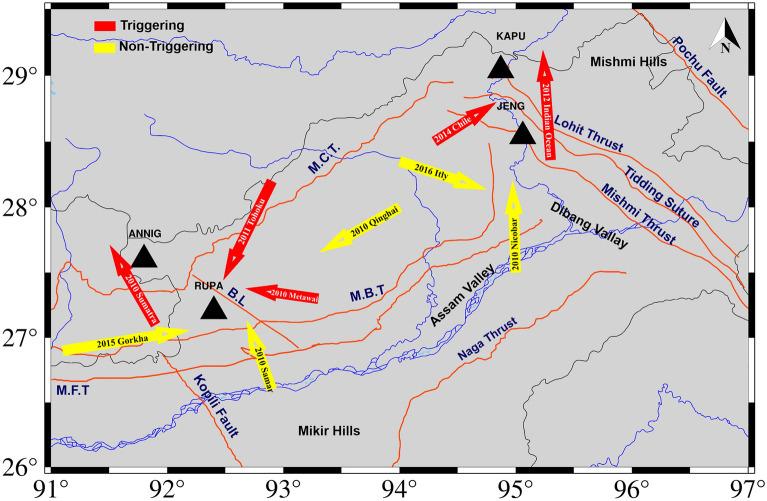
Plot of incoming seismic waves at the triggered stations ANNIG, RUPA, KAPU, and JENG stations with respect to faults. The length of the arrows is proportional to the amplitude of the incoming surface waves, which is calculated as sqrt [(amplitude of Transverse)^2^ + (amplitude of Radial)^2^]. The triggered and non-triggered teleseismic events (having dynamic stress ≥ 10 kPa) are shown by the red and yellow arrows. The Seismotectonic data used for plotting the faults (solid orange line), Geological Survey of India, Government of India, Kolkata, India, last access on 09 October 2023, https://bhukosh.gsi.gov.in/Bhukosh/Public. The figure is made using GMT version 6.3.0.

Many recent studies have shown that the direction of incoming waves could be a function of dynamic triggering^22,51,52^ and have documented that the incidence angle of the triggering waves is parallel to the strike of the central ridge in Taiwan. Earlier studies identified that the faults in NE Iran parallel to the incoming waves are most likely to experience triggering^15^. Moreover, similar results have been found in the Coso Geothermal Region^53^. Our finding is consistent with the aforementioned observations. Hence, the incidence of the incoming surface waves relative to the faults is significant in the Arunachal Himalaya (Fig. 11). The events of Sumatra 2010, Mentawai 2010, and the Indian Ocean 2012 formed an angle of ~ 120° with regard to the fault located near the respective recording station. In a similar manner, the seismic events in Tohoku 2011 and Chile 2014 were shown to have a relative angle of ~ 60° with respect to the fault. Therefore, angles ~ 60° and ~ 120° play an important role in triggering (Fig. 11).

The back-azimuth angle (BAZ) is important in understanding how stress is conveyed. When seismic waves from a teleseismic event contact local geological features at a particular angle, they may cause stress to be transferred to neighbouring fault lines. The highest stress encountered during this interaction, represented by the peak dynamic stress, might increase the chance of local earthquakes being produced^15^. In our study, the triggering has been identified at various back azimuth angles (red circles) with respect to peak dynamic stress. We do not get a particular angle for the triggering (Supplementary Fig. S5). The highest dynamic stress was seen at an angle of 270°; however, no indication of triggering was observed at this specific angle. Therefore, it may be inferred that the back azimuth (BAZ) is an improbable factor for triggering in the region.

Another possible reason for triggering is subcritical crack growth. Dynamic stress is intimately related to subcritical crack growth, a progressive process where microscopic cracks inside a geological fault zone expand as a result of chemical interactions between different rock types and water^54^. The vibrations caused by wave energy act as a rapid jolt, stressing the cracks. These little cracks may start to spread because of the dynamic stress caused by seismic waves. Following the rules of subcritical fracture development, the cracks grow larger, which increases the stress at their points. The increased stress speeds up the pace at which these cracks expand, maybe to the point where they become large enough to cause a seismic event. Fundamentally, the interaction between dynamic forces and subcritical fracture formation creates a complicated process that may help to triggering in the region^54^.

The dynamic stress threshold that triggers earthquakes varies according to geographical locations and the tectonic environment. Many recently conducted studies have shown that the dynamic stress of 1 kPa is sufficient to cause seismicity worldwide^55,56^. The dynamic stress range of 4–11 kPa can trigger the tremor in Japan. If we consider the case of the Himalayas we already discussed, there are only a few studies^20,26,27^ related to dynamic triggering. In the central Himalayas, the ~ 9 kPa dynamic stress triggers approximately 40 earthquakes during the first 12 h of teleseismic wave arrival^27^. In the present study we found dynamic stress of 1 kPa is capable of triggering seismicity in this region.

## Conclusion

A systematic investigation was conducted to examine dynamic triggering in Arunachal Himalaya using 34 significant remote and distant earthquakes between April 2010 and December 2018, having peak dynamic stress of at least 1 kPa. Using the waveform data, we identified triggered seismicity in the form of microearthquakes and non-volcanic tremors during six remote mainshocks.The triggering are not significantly influenced by the BAZ. The angle (~ 60° and ~ 120°) of incoming seismic waves with respect to the fault is a possible reason for the triggering. It is important to note that multiple processes may be occurring at the same time and contributing to the triggering in the region. The largest triggered event was M_L_2.3, 7.5 h after the occurrence of the Indian Ocean earthquake, M_w_8.6. The majority of the events with higher peak dynamic stress do not cause earthquakes or tremors. However, under some conditions, it is seen that a dynamic stress of 1 kPa is capable of triggering. We infer that the Arunachal Pradesh region is extremely stressed, and even tiny stresses can cause seismic triggering.

### Supplementary Information

Supplementary Information.

## Data Availability

Data is from an ongoing project of CSIR-NGRI, Hyderabad and available on request from NPR.
