# Supplementary material for: Tiny stresses are capable of triggering earthquakes and tremors in Arunachal Himalaya
Source: Sci Rep. 2023 Dec 14;13:22223. doi: 10.1038/s41598-023-49068-3 (PMC10721823; doi:10.1038/s41598-023-49068-3)
Supplement: Supplementary file 1 — Supplementary Information. [file 41598_2023_49068_MOESM1_ESM.docx]

**Tiny stresses are capable of triggering earthquakes and tremors in Arunachal Himalaya**

ST: Stations used in study

| S. No. | Station Name | Latitude | Longitude |
| --- | --- | --- | --- |
| 1 | ANNIG | 27.600 | 91.8528 |
| 2 | BAGR | 28.4638 | 92.1099 |
| 3 | BAIS | 27.4638 | 92.1099 |
| 4 | BARI | 27.946 | 94.4462 |
| 5 | BENE | 28.1979 | 94.7242 |
| 6 | BICH | 27.3047 | 92.6166 |
| 7 | DEED | 27.5895 | 93.6761 |
| 8 | DIRA | 27.3578 | 92.2373 |
| 9 | DITE | 28.3624 | 95.0706 |
| 10 | ELEP | 27.0931 | 92.5892 |
| 11 | JENG | 28.547 | 95.061 |
| 12 | KAPU | 29.0406 | 94.8747 |
| 13 | KAYI | 28.4018 | 94.6882 |
| 14 | KODA | 28.2414 | 94.115 |
| 15 | KOLO | 27.9045 | 93.3529 |
| 16 | MENG | 28.0999 | 94.1462 |
| 17 | MIGN | 28.8502 | 94.7834 |
| 18 | NARI | 27.38 | 93.04 |
| 19 | NAVA | 28.3615 | 93.6631 |
| 20 | PAKE | 27.5611 | 92.9754 |
| 21 | PLIN | 27.6888 | 93.6298 |
| 22 | PANG | 28.2160 | 94.9999 |
| 23 | POTN | 27.3366 | 93.8225 |
| 24 | PUCH | 27.8434 | 94.1674 |
| 25 | RAMS | 28.6584 | 95.002 |
| 26 | RILU | 27.8505 | 94.7933 |
| 27 | RUPA | 27.2040 | 92.4012 |
| 28 | SEPA | 27.3422 | 93.0394 |
| 29 | SRLI | 27.9523 | 93.1627 |
| 30 | SGRM | 27.81 | 93.5324 |
| 31 | TABA | 27.9599 | 94.332 |
| 32 | TATO | 28.5256 | 94.369 |
| 33 | TAWG | 27.6028 | 91.857 |
| 34 | ZIRO | 27.5315 | 93.7788 |


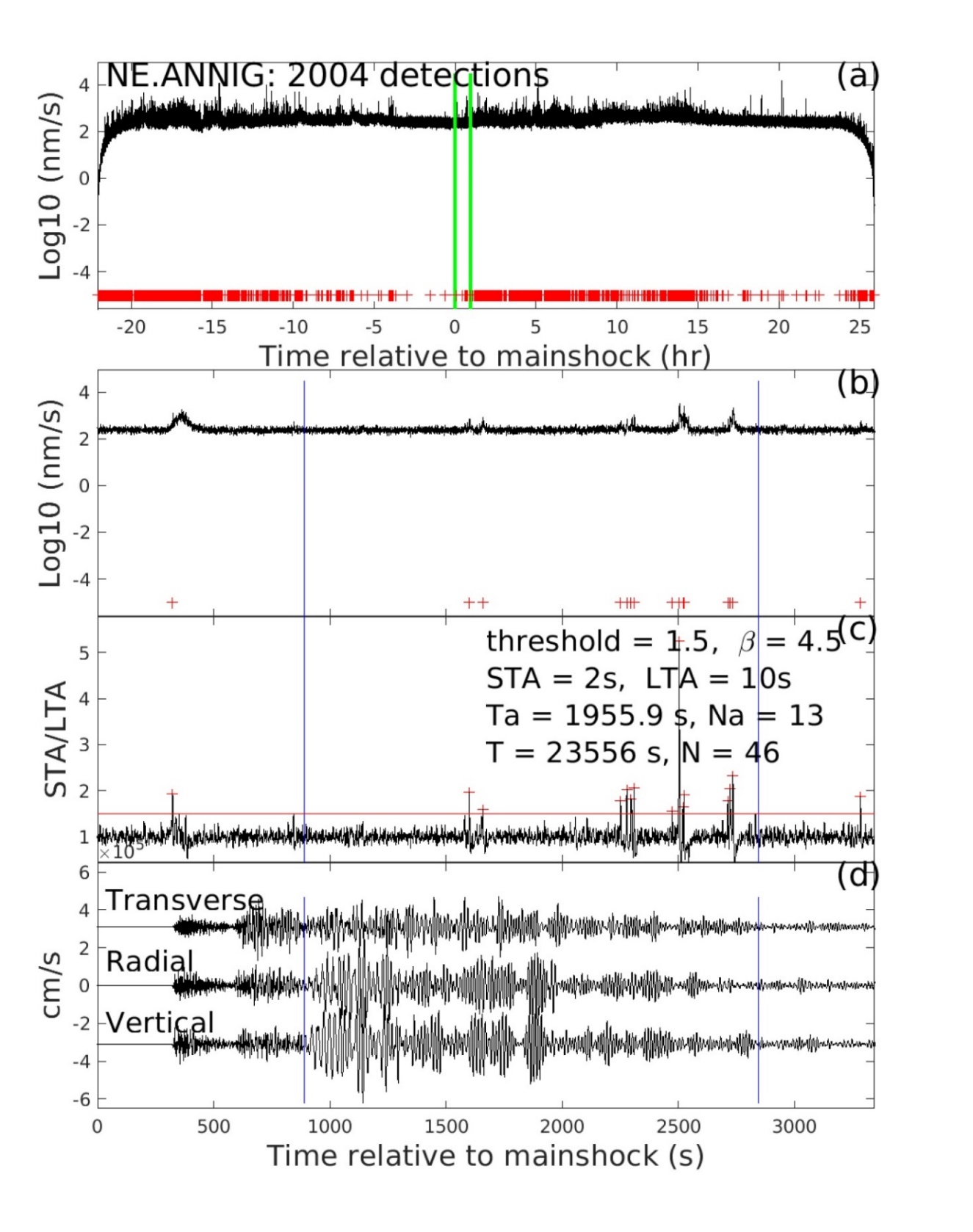


**Fig. S1** β Value computation at the ANNIG station during the Sumatra Earthquake on April 6, 2010, Mw7.8 (a) Log 10 of 5 Hz passed averaged of transverse, radial, and vertical component envelope, (b) plot of the green vertical lines in panel (a), (c) the ratio of the Short-Term Average (STA) to Long-Term Average (LTA). (d) Unfiltered waveform with transverse, radial, and vertical component portions. The red markers in (b) and (c) indicate the position of peaks in the envelope. The blue vertical lines indicate the duration of the surface waves. The β value of 4.5 indicates a significant increase in the seismicity during the passage of surface waves.


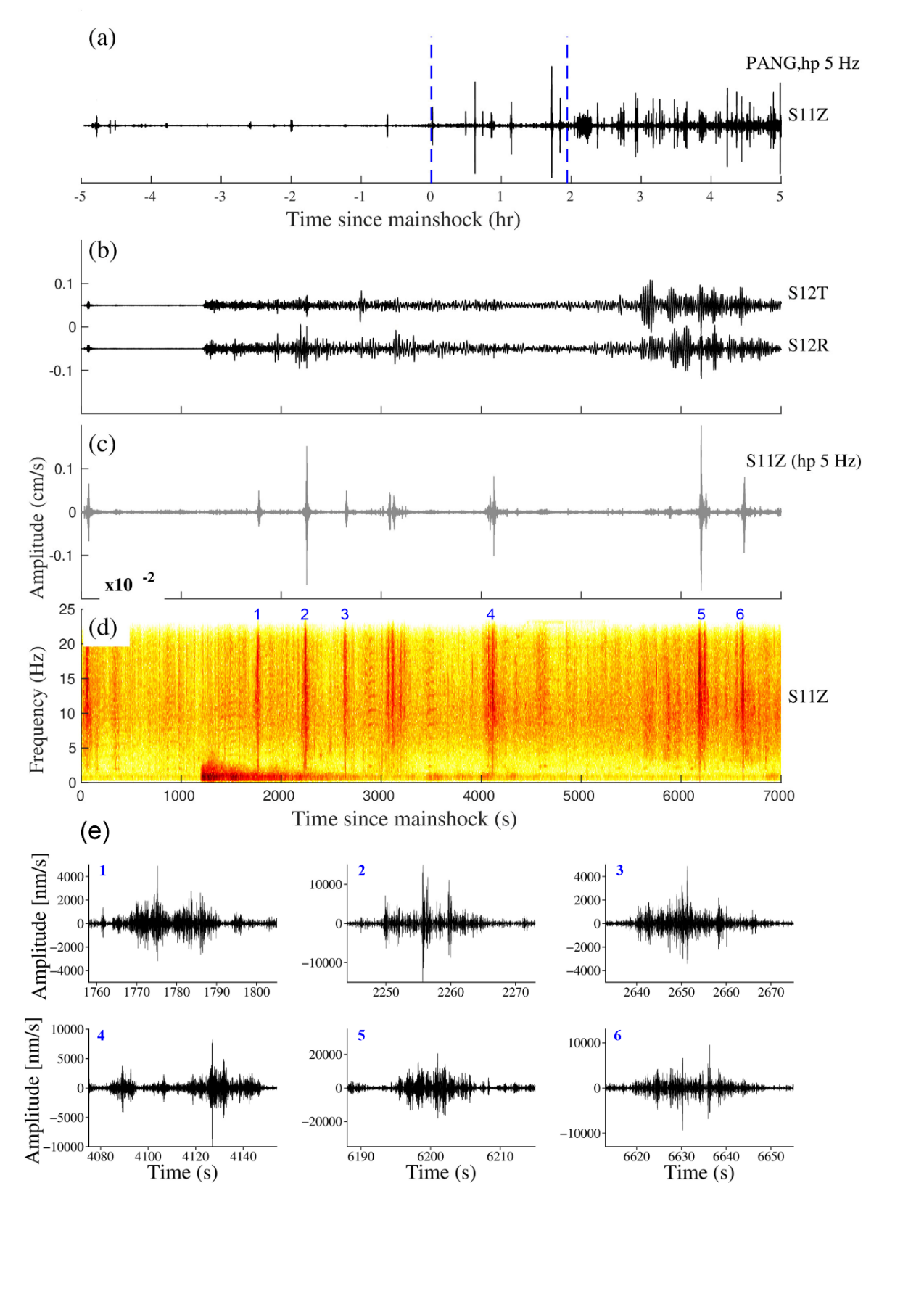


**Fig. S2** (a) Vertical components of the 5 Hz band passed waveform at PANG station during the Iquique earthquake, April 1, 2014, Mw 8.2, (b), (c) and (d) are Zooming portion of the blue vertical line in (a), (b) Transverse and Radial component, (c) Vertical component with a 5 Hz high pass filter, (d) Vertical component spectrogram, (e) zoom in the portion of the number marked on the spectrogram and black colour waveform represent (high pass 5 Hz) triggered tremors. (b) and (c) follow the same time scale of (d).


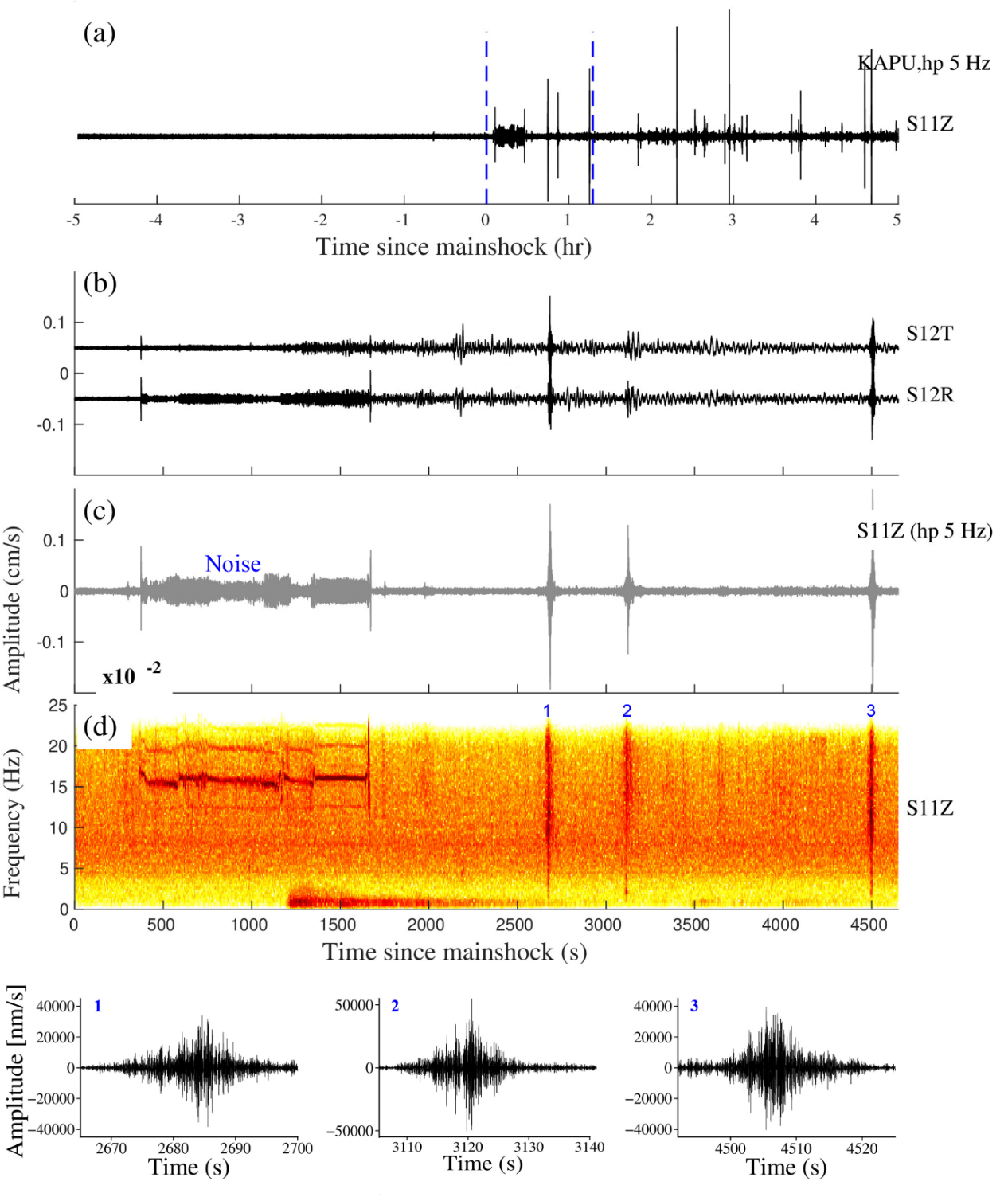


**Fig. S3** (a) Vertical components of the 5 Hz band passed waveform at KAPU station during the Iquique earthquake, April 1, 2014, Mw 8.2, (b), (c) and (d) are Zooming portion of the blue vertical line in (a), (b) Transverse and Radial component, (c) Vertical component with a 5 Hz high pass filter, (d) Vertical component spectrogram, (e) zoom in the portion of the number marked on the spectrogram and black colour waveform represent (high pass 5 Hz) triggered tremors. (b) and (c) follow the same time scale of (d).


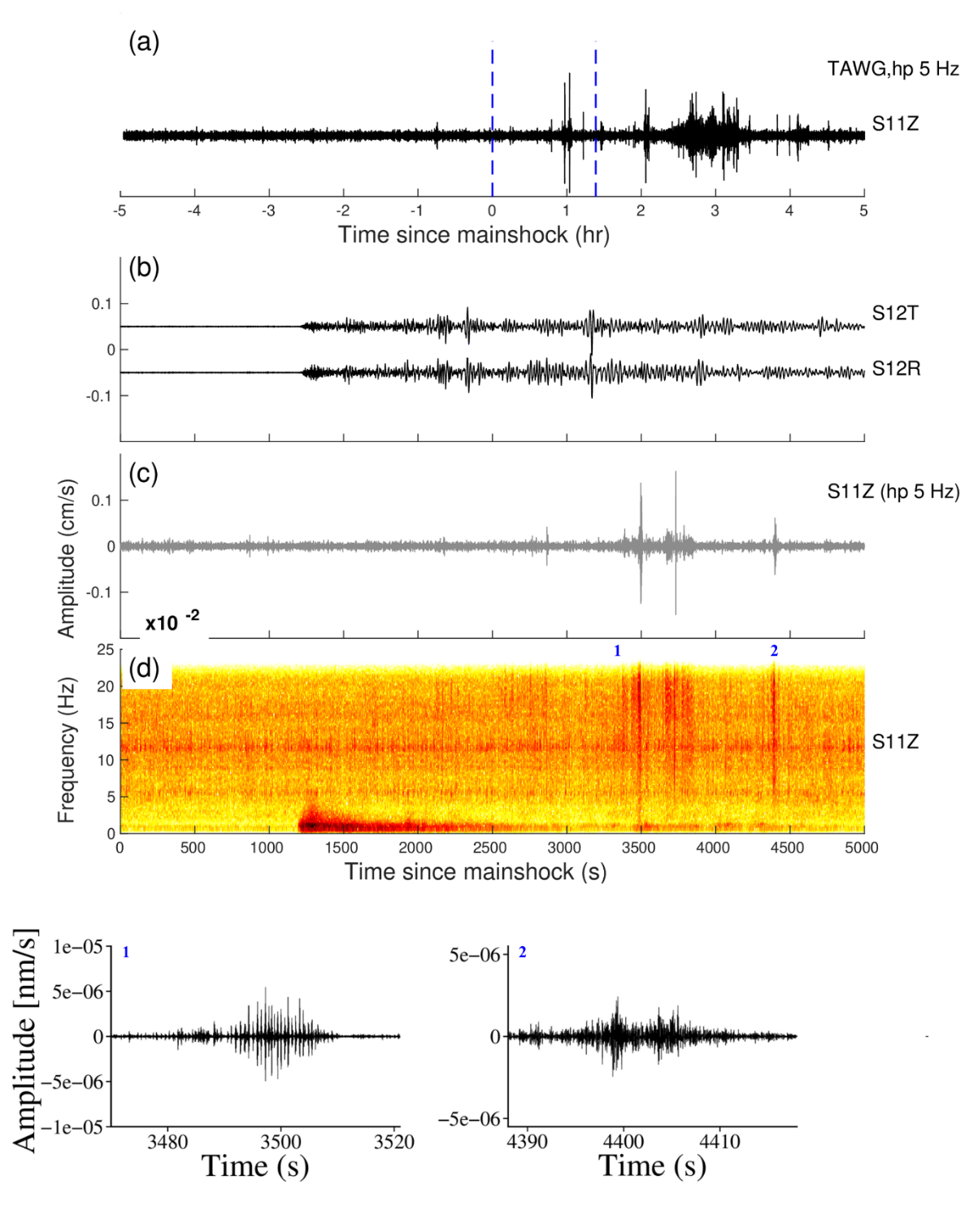


**Fig. S4** (a) Vertical components of the 5 Hz band passed waveform at TAWG station during the Iquique earthquake, April 1, 2014, Mw 8.2, (b), (c) and (d) are Zooming portion of the blue vertical line in (a), (b) Transverse and Radial component, (c) Vertical component with a 5 Hz high pass filter, (d) Vertical component spectrogram, (e) zoom in the portion of the number marked on the spectrogram and black colour waveform represent (high pass 5 Hz) triggered tremors. (b) and (c) follow the same time scale of (d).


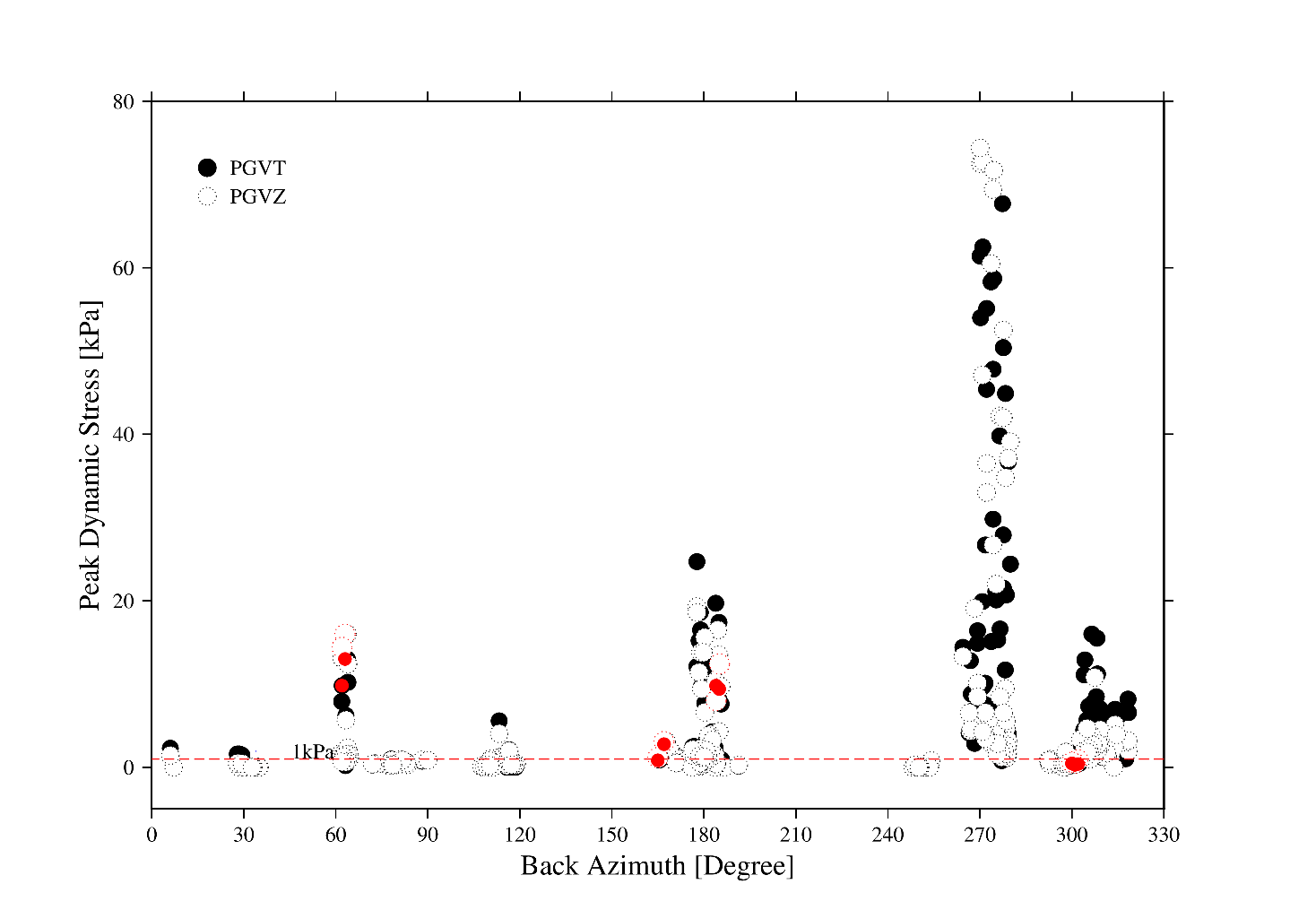


**Fig. S5** Dynamic stresses at vertical (open circle) and transverse (close circle) of all analysed earthquakes versus back azimuth angle. PGVT and PGVZ stands for Peak Ground Velocity at Transverse and Peak Ground Velocity at vertical (Z) component. The triggered events are represented by red open and closed circles. Red solid and open circle represent the peak ground velocity of transverse and radial component of triggered events. The red dotted horizontal line represents a dynamic stress of 1 kPa.


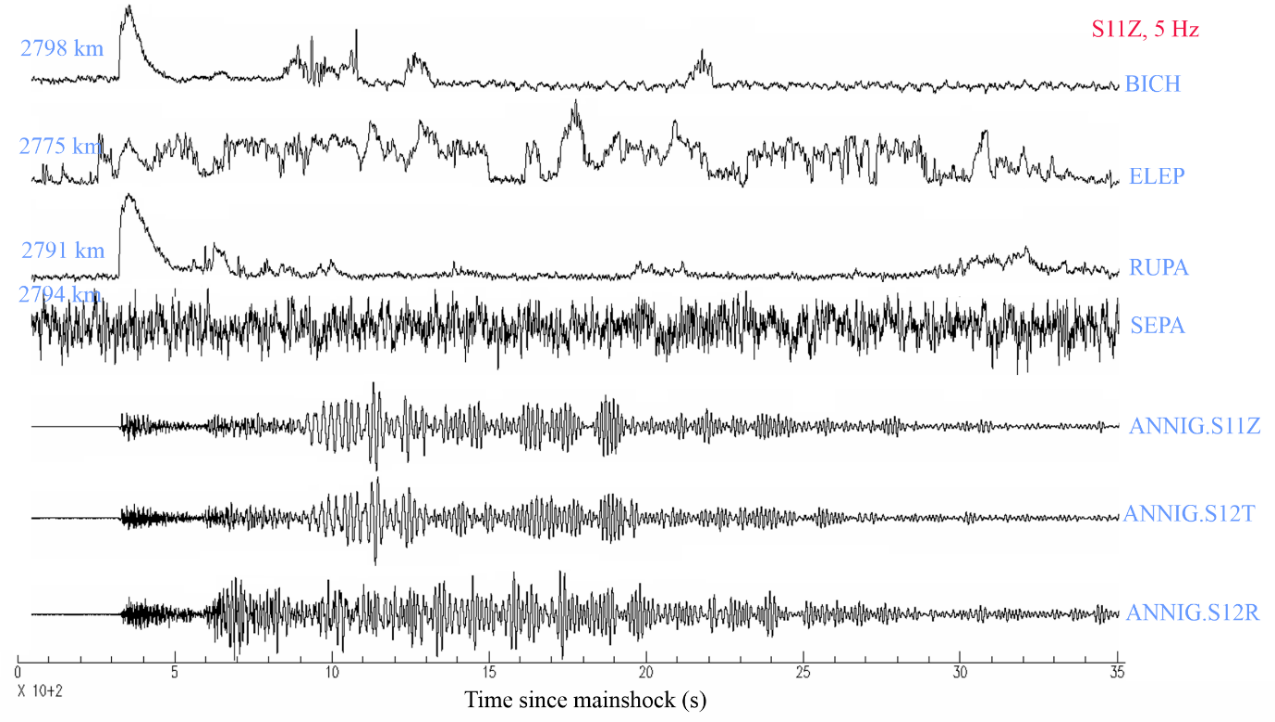


**Fig. S6** 5HZ high passed Log envelope function of the vertical component at BICH, ELEP, RUPA, SEPA stations during the April 6, 2010, Mw 7.8 Sumatra earthquake. In the lower panel unfiltered waveform at ANNIG station is plotted. There is no triggering found during the event. The peaks in the envelope are due to the noise.


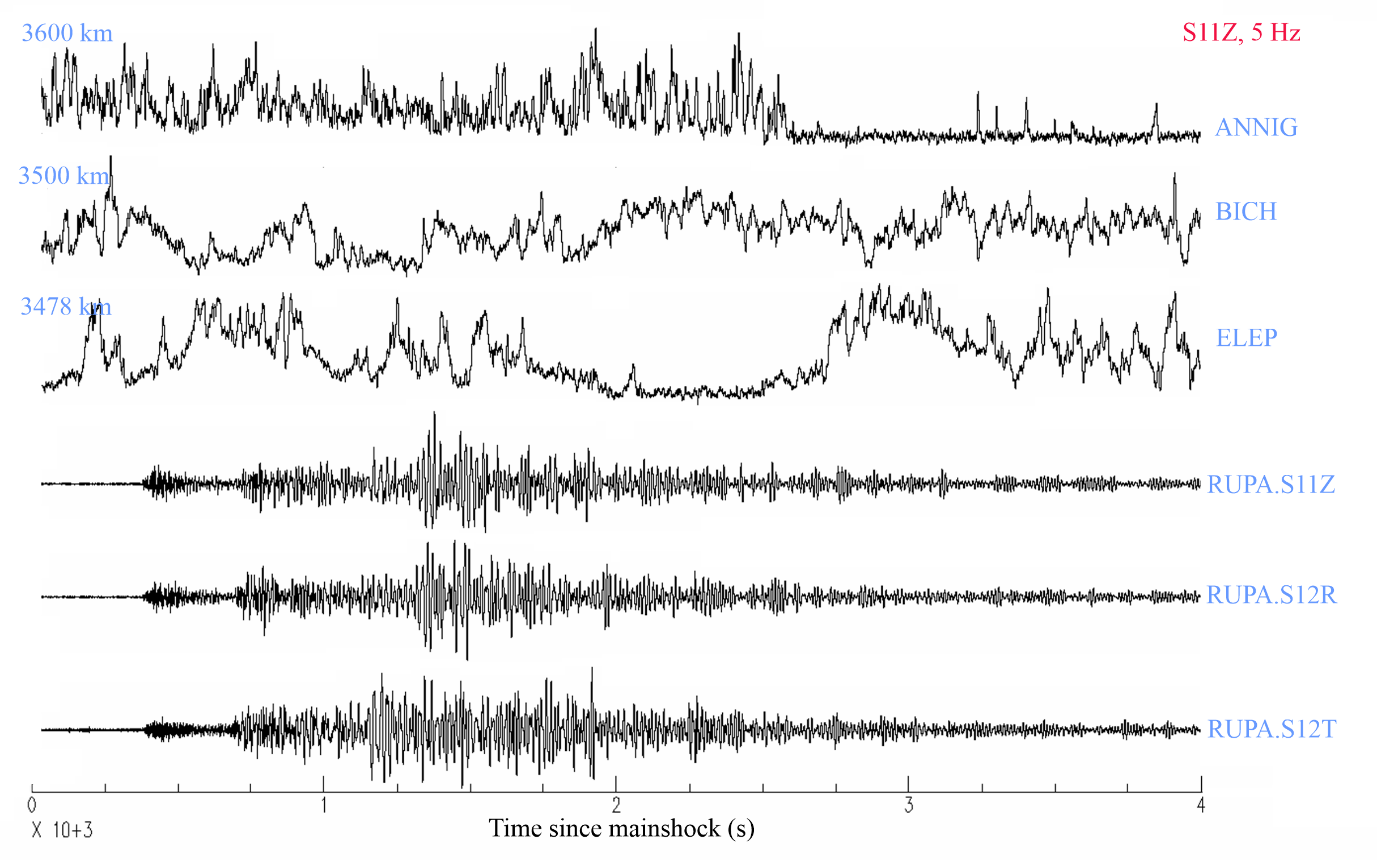


**Fig. S7** 5HZ high passed Log envelope function of the vertical component at ANNIG, BICH, ELEP stations during the October 25, 2010, Mw 7.8 Mentawai earthquake. In the lower panel unfiltered waveform at RUPA station is plotted. There is no triggering found during the event. The peaks in the envelope are due to the noise.


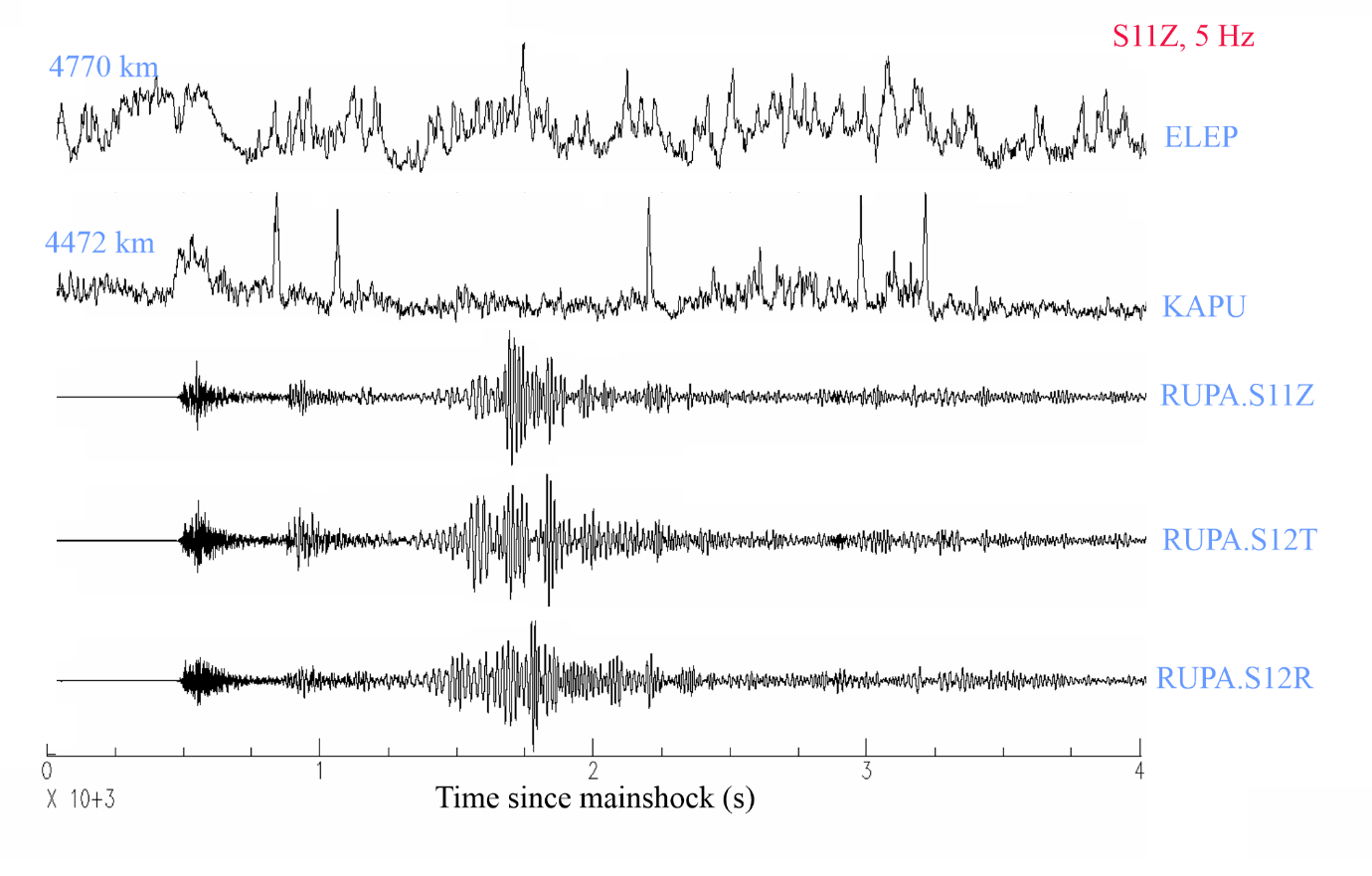


**Fig. S8** 5HZ high passed Log envelope function of the vertical component at ELEP, KAPU stations during the March 11, 2011, Mw 9.1 Tōhoku earthquake. In the lower panel unfiltered waveform at RUPA station is plotted. There is no triggering found during the event. The peaks in the envelope are due to the noise.


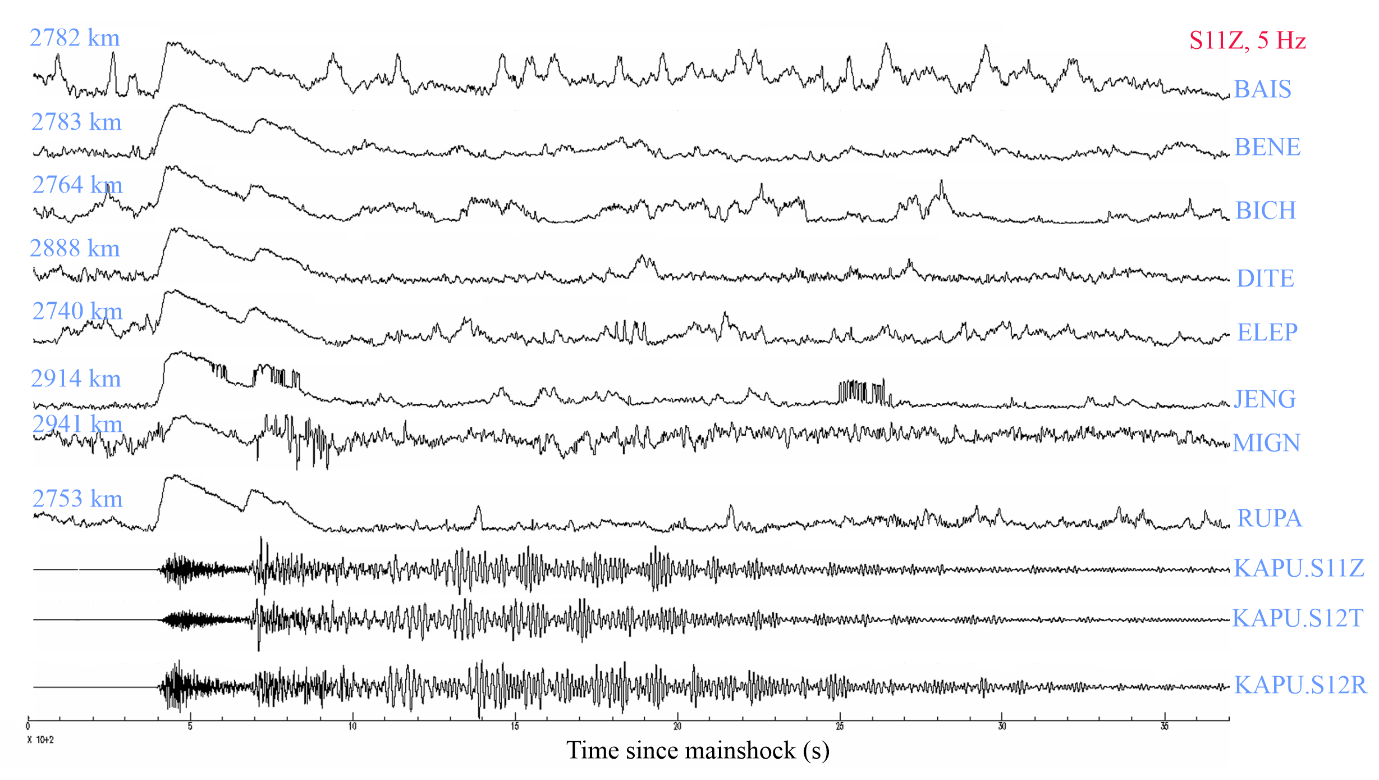


**Fig. S9** 5HZ high passed Log envelope function of the vertical component at BAIS, BENE, BICH, DITE, ELEP, JENG, MIGN, RUPA stations during the April 11, 2012, Mw 8.6 Indian Ocean earthquake. In the lower panel unfiltered waveform at KAPU station is plotted. There is no triggering found during the event. The peaks in the envelope are due to the noise.


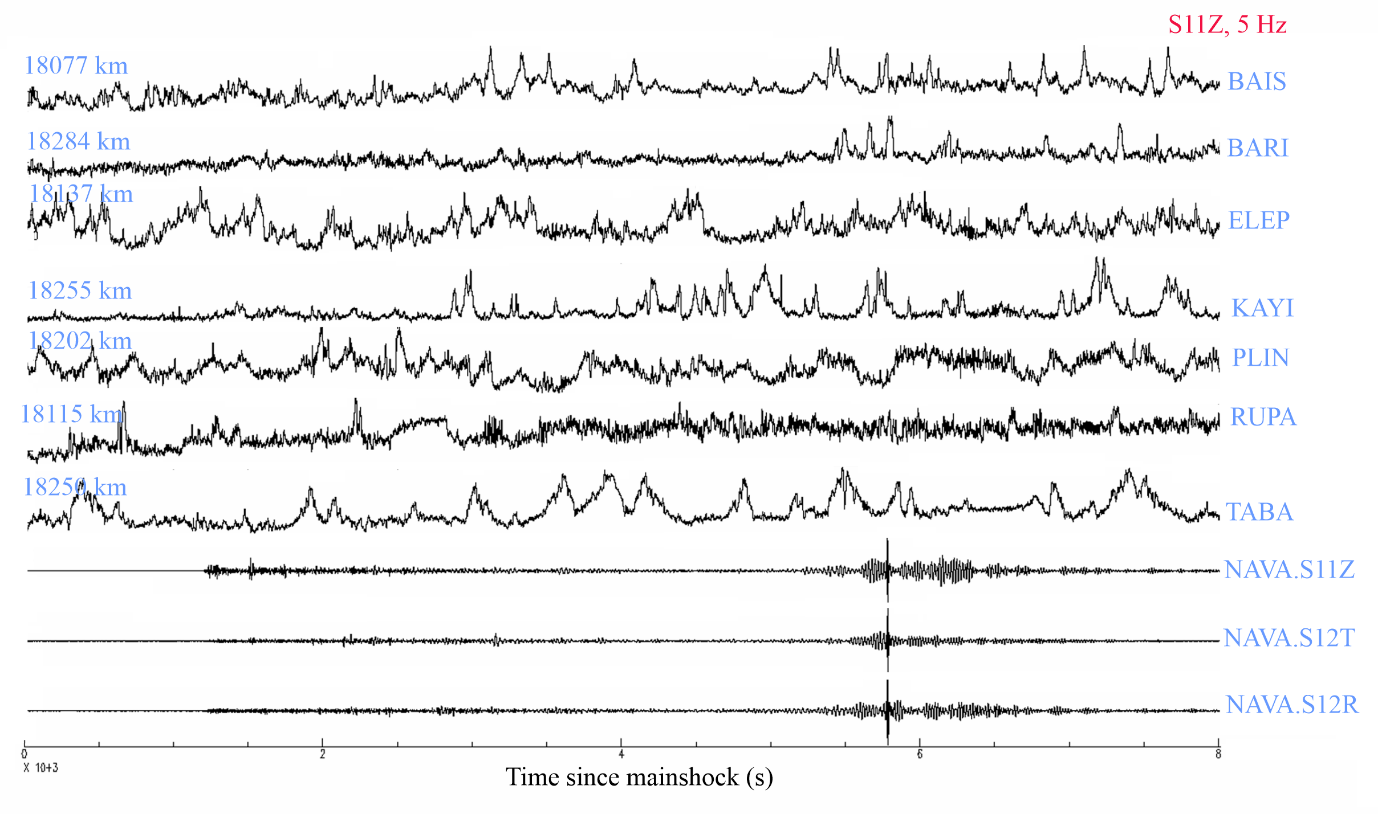
**Fig. S10** 5HZ high passed Log envelope function of the vertical component at BAIS, BARI, ELEP, KAYI, PLIN, RUPA, TABA stations during the April 1, 2014, Mw 8.2 Iquique earthquake. In the lower panel unfiltered waveform at NAVA station is plotted. There is no triggering found during the event. The peaks in the envelope are due to the noise.
